# Clinical and social factors associated with involuntary psychiatric hospitalisation in children and adolescents: a systematic review, meta-analysis, and narrative synthesis

**DOI:** 10.1016/S2352-4642(21)00089-4

**Published:** 2021-07

**Authors:** Susan Walker, Phoebe Barnett, Ramya Srinivasan, Esha Abrol, Sonia Johnson

**Affiliations:** a Division of Psychiatry, University College London, London, UK; b Department of Clinical Educational and Health Psychology, Centre for Outcomes Research and Effectiveness, University College London, London, UK; c National Institute of Health Research Mental Health Policy Research Unit, University College London, London, UK; d Great Ormond Street Institute of Child Health, University College London, London, UK; e Camden and Islington NHS Foundation Trust, London, UK

## Abstract

**Background:**

Disparities in involuntary psychiatric hospitalisation between population subgroups have been identified in adults, but little is known about the factors associated with involuntary hospitalisation in children or adolescents. We did a systematic review, meta-analysis, and narrative synthesis to investigate the social and clinical factors associated with involuntary psychiatric hospitalisation among children and adolescents.

**Methods:**

We searched MEDLINE, PsycINFO, Embase, and the Cochrane Central Register of Controlled Trials for studies of any type up to July 22, 2020, that compared the characteristics of voluntary and involuntary psychiatric inpatients (mean age of sample ≤18 years). We synthesised results using random effects meta-analysis on unadjusted data and by narrative synthesis. Heterogeneity between studies was calculated using *I*^2^. This study is registered on PROSPERO, CRD42020099892.

**Findings:**

23 studies from 11 countries were included in the systematic review and narrative synthesis, of which 19 studies (n=31 212) were included in the meta-analysis. On meta-analysis, involuntary rather than voluntary hospitalisation of minors was associated with a diagnosis of psychosis (eight studies; odds ratio 3·63, 95% CI 2·43–5·44, p<0·0001), substance misuse (five studies; 1·87, 1·05–3·30, p=0·032), or intellectual disability (four studies; 3·33, 1·33–8·34, p=0·010), as well as presenting with a perceived risk of harm to self (eight studies; 2·05, 1·15–3·64, p=0·015) or to others (five studies; 2·37, 1·39–4·03, p=0·0015). Involuntary hospitalisation was also found to be associated with being aged 12 years or older (three studies; 3·57, 1·46–8·73, p=0·0052) and being from a Black rather than a White ethnic group (three studies; 2·72, 1·88–3·95, p<0·0001). There was substantial between-study heterogeneity for most factors included in the meta-analysis (*I*^2^ from 51·3% to 92·3%). Narrative synthesis found that more severe illness and poorer global functioning was associated with involuntary hospitalisation.

**Interpretation:**

Over-representation of involuntary psychiatric hospitalisation in certain groups might begin in childhood, potentially establishing a cycle of inequality that continues into adulthood. Further research into the systemic factors underlying these health-care inequalities and the barriers to accessing less coercive psychiatric treatment is urgently required, with specific consideration of racial and ethnic factors.

**Funding:**

UK National Institute for Health Research and Wellcome Trust.

## Introduction

The 2018 UK independent review of the Mental Health Act 1983 recognises that the use of involuntary psychiatric hospitalisation can “help restore health, and even be life-saving”, but is also potentially “traumatic, frightening and confusing”,^1^ and represents a centuries-old debate about society's need to balance paternalism with autonomy. Involuntary hospitalisation is generally used as a last resort and is designed to offer protection to those who are temporarily unable to protect themselves or those around them due to the presence of a mental disorder. Although mental health legislation differs internationally and even intranationally, an involuntary hospitalisation is authorised only when specific legal criteria are met. In most European countries, these criteria include presenting with a broadly defined mental disorder and risk to oneself or others.^2^ Involuntary hospitalisation also usually confers additional protections, such as the right to appeal and mandatory post-discharge care. However, involuntary treatment is sometimes experienced as traumatic,^3^ can lead to future reluctance to engage with mental health care,^4^ and can be associated with other restrictive interventions such as seclusion and restraint.^5^ In addition, growing evidence indicates that factors outside of those specified in mental health legislation can affect and potentially systematically bias decisions around who needs involuntary treatment.^6^, ^7^, ^8^

In adults, people from Black and minority ethnic groups are more likely to be hospitalised against their will than people from White and non-minority groups.^7^ Other sociodemographic factors associated with involuntary care of adults are male gender, unemployment, receiving welfare benefits, and living in areas of increased deprivation.^6^ Additionally, people with a diagnosis of psychosis, those brought into hospital by police, and those who have been hospitalised involuntarily before are more likely to have an involuntary than a voluntary admission.^6^ However, little is known about the social and clinical factors that could increase the likelihood of an involuntary psychiatric hospitalisation among children and adolescents. Given that a previous involuntary hospitalisation is associated with future involuntary hospitalisation, an involuntary hospitalisation in childhood or adolescence might increase the risk of further coercive care in adulthood, potentially establishing a cycle of health-care inequalities and increased use of coercive treatment among certain groups.

**Research in context**

**Evidence before this study**

We did preliminary searches of MEDLINE, PsycINFO, and Embase between January, 1983, and May, 2018, with no restriction by language, as a scoping review. Our search terms included “mental health” OR “involuntary treatment” OR “psychiatric hospitalisation” AND “risk factor”, and we limited the search to studies of individuals younger than 18 years. We identified few studies on children and adolescents and involuntary hospitalisation and no systematic reviews or meta-analyses on this topic.

**Added value of this study**

Previous research into the factors associated with involuntary psychiatric hospitalisation has focused on adult populations. Some adults, such as those from Black and minority ethnic groups and those who have been detained before, are more likely to have an involuntary than a voluntary psychiatric admission, but the reasons for these differences remain unclear. Based on a small number of studies, we identified that involuntary rather than voluntary psychiatric hospitalisation among children and adolescents was associated with older age (12 years or older), a diagnosis of psychosis, substance misuse, intellectual disability, and presenting as a risk to oneself or others. We also found that young people from crudely defined Black ethnic groups were more likely to be hospitalised involuntarily than were young people from White ethnic groups.

**Implications of all the available evidence**

The over-representation of certain groups in involuntary care might begin in childhood and establish cycles of health inequality that persist into adulthood. Understanding the social and clinical factors associated with involuntary hospitalisation among individuals younger than 18 years has received little academic, clinical, or political attention to date, but is essential in order to address causes and pathways of detention; identify targets for interventions to reduce the use of coercive practice; and prevent the establishment of potentially lifelong negative mental health treatment trajectories.

The small amount of research on the involuntary hospitalisation of children and adolescents to date could be because mental health legislation is used less often to detain them against their will than adults.^1^ However, the involuntary hospitalisation of children and adolescents is increasing in the UK, and has also been increasing in other countries, although up-to-date data are scarce.^9^, ^10^, ^11^, ^12^ For example, in Finland, involuntary admissions of people younger than 18 years was 2·4 per 10 000 in 1995, and increased to 7·2 per 10 000 in 2000.^10^ In addition, although in most countries the essential legal criteria for the involuntary hospitalisation of children and adolescents are the same as for adults,^13^ the hospitalisation of children and adolescents is complicated by the role of parents and guardians. In the UK, for example, a person aged 15 years or younger with a mental disorder who does not want to be admitted can, according to the law, be admitted to hospital under parental consent, and would legally be defined as a voluntary patient.^14^ If this same young person's parents did not consent to the admission (or potentially one parent did and the other did not), they could be admitted to hospital involuntarily, under the Mental Health Act. The difference between these two scenarios is the views of the parents and not necessarily the needs of the young person. Equally, a 16-year-old in the UK who agrees to be admitted might be thought not to have capacity to consent to this because of their developmental level and can be admitted to hospital involuntarily under the Mental Health Act. Therefore, a binary distinction between voluntary and involuntary hospitalisation of young people might be overly simplistic. However, understanding more about the clinical and sociodemographic factors that are associated with legally defined involuntary and voluntary admissions of young people is important in order to design early interventions to reduce coercion; to ensure equity of care; and to potentially prevent negative service trajectories being established.

To our knowledge, no previous systematic reviews or meta-analyses of the factors associated with involuntary psychiatric hospitalisation among children and adolescents have been done. We aimed to assess international evidence on the associations between social and clinical factors and the involuntary hospitalisation of children and adolescents.

## Methods

### Search strategy and selection criteria

This systematic review and meta-analysis adhered to the PRISMA guidelines.^15^ We included quantitative studies published in peer-reviewed journals that recorded patients in or admitted to psychiatric hospital voluntarily and involuntarily. In line with the UK National Health Service long-term plan that youth mental health services should cover individuals aged up to 25 years, study samples that included people aged up to 25 years were included if the mean age of the sample was 18 years or younger. The primary outcome of interest was involuntary psychiatric hospitalisation under mental health law, and patients hospitalised voluntarily were the comparison group. Given the lack of research in the field, all types of research study were considered, including cross-sectional and cohort studies. Studies were also included in the narrative synthesis if they met the inclusion criteria but did not contain data that could be used in the meta-analysis.

The search strategy was adapted from the strategy we developed previously to look at social and clinical factors associated with involuntary hospitalisations among adults.^6^ We searched MEDLINE, PsycINFO, Embase, and the Cochrane Central Register of Controlled Trials using keyword and subject headings from database inception to Aug 31, 2019, and updated the search on July 22, 2020. We did not restrict our search by language. We supplemented the search strategy with a backwards reference search of included studies and any relevant reviews, and a forward citation search using Scopus. Full search strategies are available in the appendix (pp 1–3).

One reviewer (SW) identified studies that met inclusion criteria through systematic screening of all titles and abstracts, then the full text. At each stage, a random 10% check was done by an independent second reviewer (RS). Any discrepancies were resolved by consensus and discussion with a senior reviewer (SJ).

### Data extraction and quality assessment

Two of the authors (SW and RS) extracted data independently using a Microsoft Excel-based broad extraction sheet, which included study design, sample size, country, diagnosis, age, gender, ethnicity, where and with whom the young people were living, previous abuse, socioeconomic status, educational level, risk to self and others, pathways to care, and our primary outcome measures (the number of young people admitted voluntarily and involuntarily). These factors had been identified in advance through our scoping review and expert consultation, but we also extracted data on any other factors associated with involuntary hospitalisation that were identified in the individual studies.

Three reviewers (SW, RS, and EA) assessed the quality of included studies using the 14-item checklist developed by Kmet and colleagues,^16^ a tool suitable for use with a range of study designs. Every study was assessed against each of the 14 items using a 3-point scale, with a score of 2 showing that criteria were fully met, a score of 1 denoting that criteria were partly met, and a score of 0 showing that criteria were not met. A linear summary score (total sum divided by total possible sum) from 0 to 100 was calculated and each study was then categorised as low (≤49), moderate (50–74), or high (≥75) quality. Scores for each study are available in the appendix (p 13).

In line with the methodology from previous studies in this field,^6^, ^7^ 10% of the study extraction and quality assessment were independently checked by two reviewers (RS and SW). Given the low numbers of studies involved, it was not possible to calculate inter-rater reliability scores. There were discrepancies about the data extraction for two of the papers, but these were resolved through discussion. In the quality assessment, there were no discrepancies in the final summary scores. Any discrepancies would have been resolved through further checks and discussion with a senior reviewer (SJ).

### Data analysis

We used Comprehensive Meta-Analysis software (version 3)^17^ and the metafor package in the statistical program R (version 4.0.2)^18^ to calculate random effects summary estimates (odds ratios [ORs] and 95% CIs) for the association between the ten meta-analysable variables (gender, primary diagnosis, ethnicity, living arrangements, risk of harm to self, risk of harm to others, previous abuse, previous psychiatric hospitalisation, age, and intellectual disability) and involuntary hospitalisation. Only unadjusted data were included in our meta-analyses. Post-hoc meta-regressions to assess possible causes of heterogeneity were planned, but in line with Cochrane Handbook guidance, only if there were ten or more studies for each variable.^19^

Intellectual disability is not traditionally classed as a psychiatric disorder (due to its early onset and pervasive nature). In the International Classification of Disorders (ICD) and the Diagnostic and Statistical Manual of Mental Disorders (DSM) multiaxial systems (used before DSM-5), it is treated as a developmental disorder (Axis II) rather than a psychiatric disorder (Axis I). In addition, the UK Mental Health Act code of practice states that “a person must not be considered to be suffering from a mental disorder solely because they have a learning disability”.^14^ As such, we included all young people with a diagnosis of intellectual disability in the meta-analysis, whether this was described as the main diagnosis or a comorbid one. All of the other diagnostic categories included in the meta-analysis were based on the main or primary diagnosis only, with studies excluded from the meta-analysis if multiple diagnoses were given per patient.

We calculated heterogeneity between studies using *I*^2^. A value of 0% indicates no observed heterogeneity, 25% indicates low heterogeneity, 50% indicates moderate heterogeneity, and 75% indicates high heterogeneity.^20^ We assessed publication bias by visual examination of the funnel plot.

The narrative synthesis was done following guidance for systematic reviews.^21^ We identified factors in the broad extraction sheet that were not suitable for a meta-analytic approach because they were not reported consistently or with the necessary data. These included psychiatric symptomatology, associations between gender and diagnosis, previous outpatient treatment, referral pathway, family factors or living arrangements, and socioeconomic status. To synthesise all of these factors, two reviewers (SW and RS) tabulated the data by study and included a textual description of the identified factors, and whether the direction of the association with involuntary hospitalisation was positive or negative. We then regrouped data by factor of interest to investigate how each factor was associated with involuntary care across all studies.

This review was prospectively registered on PROSPERO, CRD42020099892.

### Role of the funding source

The funder had no role in study design, data collection, data analysis, data interpretation, or writing of the report.

## Results

Our initial search identified 3358 potentially eligible studies, of which 555 were identified as duplicates, resulting in 2803 studies to be screened. After screening of titles and abstracts, 101 potentially relevant full-text articles were identified, of which 22 met inclusion criteria. The updated search on July 22, 2020, identified one additional study meeting inclusion criteria (figure). No further studies were identified on the forward or backward searches.

**Figure:**
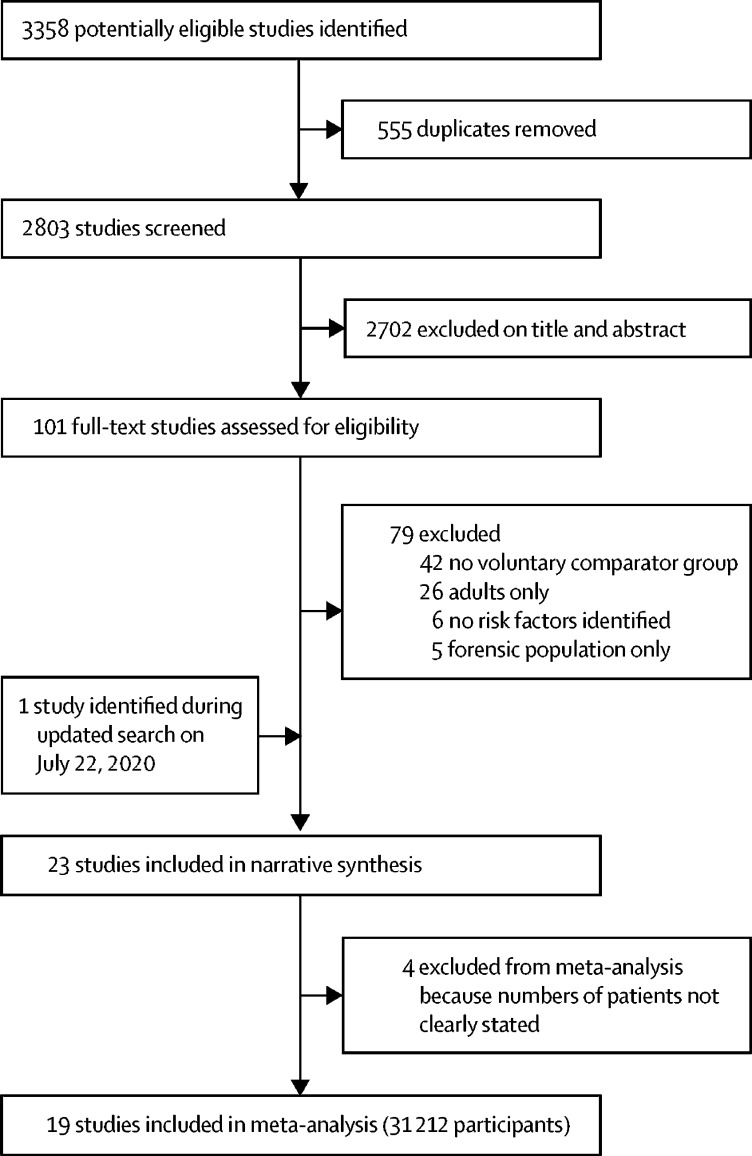
Study selection

The key characteristics of the 23 included studies are shown in table 1. The studies were all from high-income countries, with 17 from seven European countries (Finland, Germany, UK, Netherlands, Switzerland, Belgium and Sweden), two each from the USA and Canada and one each from New Zealand and Israel. In all except one study, the maximum age of the participants was 18 years.^32^ In total, 41 271 young inpatients were represented in the studies, of whom 9753 (23·6%) were hospitalised involuntarily. 19 of 23 studies were retrospective cohort studies, which relied on routinely collected data from hospital or national databases, and samples in all studies were representative of the population of patients admitted.

**Table 1:** Key characteristics of included studies

	**Setting**	**Sample size**	**Age range, years**	**Sample description**	**Patients with involuntary hospitalisation, n (%)**	**Quality**
Ayton et al (2009)^22^	England, UK	50	14–17	All young people admitted to a specialist eating disorder unit between 2003 and 2006; voluntary patients were admitted under parental consent	16 (32%)	Moderate
Chaplin et al (2015)^23^	England, UK	151	6–17	Analysis of routinely collected data from 14 general adolescent and specialist intellectual disability inpatient units as part of a larger quality improvement project	26 (17%)	Moderate
Corrigall and Bhugra (2013)^24^	England, UK	435	12–17	All admissions to an adolescent psychiatric inpatient unit between Jan 1, 2001, and Dec 31, 2010	156 (36%)	Moderate
Ellila et al (2008)^25^	Finland	278	12–17	Point prevalence study on Jan 1, 2000, of inpatients from 64 psychiatric wards in 18 hospital districts	82 (29%)	High
Jaworowski and Zabow (1995)^26^	Israel	78	15–17	Hospital records of children and adolescents admitted to a hospital in the south of Israel between April 1, 1991, and Dec 1, 1992	14 (18%)	Low
Jendreyschak et al (2013)^27^	Germany	10 547	1–17	Retrospective analysis of hospital admission registers from three major child and adolescent psychiatry hospitals between 2004 and 2009	3081 (29%)	High
Kaltiala-Heino (2004)^10^	Finland	15 858	0–17	Retrospective study of a nationally representative discharge register between 1996 and 2000	2544 (16%)	High
Kaltiala-Heino (2010)^28^	Finland	187	11–17	Retrospective database review of admissions to the adolescent psychiatry wards of Tampere University Hospital in 2004–06	42 (22%)	High
Khenissi et al (2004)^29^	Finland	106	13–18	Retrospective review of every third patient referred involuntarily for inpatient psychiatric hospitalisation in the Unit of Adolescent Psychiatry of Turku University Hospital in 1994–2002	39 (37%)	Moderate
Kilgus et al (1995)^30^	USA	352	12–18	All adolescent admissions for psychiatric care to a state hospital in South Carolina in 1988	275 (78%)	Moderate
Laget et al (2002)^31^	Switzerland	66	13–18	Retrospective review of all inpatients in an adolescent psychiatric hospital unit in Lausanne in 1998–99	16 (24%)	Low
Lindsey et al (2010)^32^	USA	383	12–22	Retrospective patient record review of African-American young people admitted to hospital after presenting to a psychiatric emergency services centre between October, 2001, and September, 2002	300 (78%)	High
Mears et al (2003)^33^	England and Wales, UK	663	Not provided	Census of inpatients in 71 child and adolescent inpatient units on Oct 19, 1999; mean age was 17 years in the involuntary hospitalisation group and 15 years in the voluntary group	127 (19%)	Low
Mertens et al (2017)^12^	Belgium	24	13–17	Adolescent patients referred to an inpatient psychiatric unit between Sept 1, 2013, and Feb 28, 2015	12 (50%)	Low
Ottisova et al (2018)^34^	England, UK	10	5–17	Trafficked children identified from electronic health records who had been admitted to psychiatric hospital within South London and Maudsley NHS Trust as inpatients between Jan 1, 2006, and Nov 21, 2014	4 (40%)	High
Park et al (2011)^35^	New Zealand	332	12–17	Retrospective review of consecutive admissions to the general psychiatric inpatient ward in Hamilton from January, 2002, to December, 2007	204 (61%)	Moderate
Persi et al (2016)^36^	Canada	225	5–17	Retrospective chart review of all discharges between April 1, 2007, and March 31, 2008, from a child and adolescent psychiatric inpatient setting serving 26 acute care hospitals	180 (80%)	Moderate
Ramel et al (2015)^37^	Sweden	261	12–17	Retrospective review of all admissions to a child and adolescent psychiatry emergency unit in Malmo in 2011	28 (11%)	Moderate
Siponen et al (2007)^11^	Finland	9865	12–17	Retrospective register study of all adolescents admitted to Finnish psychiatry hospitals from 1996 to 2003	2333 (24%)	Moderate
So et al (2019)^38^	Netherlands	227	6–18	Registry data used to identify all psychiatric hospital admissions of children and adolescents after referral to a mobile psychiatric emergency service in two areas of the Netherlands between 2008 and 2017	90 (40%)	High
Sourander et al (1998)^39^	Finland	1014	12–17	National register of hospital discharges was used to identify all patients aged 12 to 17 years discharged from child, adolescent, or adult psychiatric hospitals in 1990 and 1993	127 (13%)	Moderate
Stein and Tanzer (1988)^40^	Canada	46	Not provided	Retrospective chart review, with follow-up, of all patients discharged from the Sunnybrook Adolescent Unit between 1977 and 1984; all of the involuntarily admitted patients (n=25) and the next patient admitted voluntarily were followed up approximately 5 years later; final sample included 23 of the involuntary group (mean age 16·7 years) and 23 of the voluntary group (mean age 16·3 years)	23 (50%)*	Low
Tolmac and Hodes (2004)^41^	England, UK	113	13–17	Cross-sectional survey of adolescents with a home address in the Greater London area who were inpatients in psychiatric units on Feb 14, 2001	34 (30%)	Moderate

* Equal numbers of patients who were admitted voluntarily and involuntarily were included as part of the study design.

11 studies were rated as moderate quality, seven were rated high quality, and five were rated low quality. There was considerable variability between the studies but one of the main areas of weakness was in the data analysis, with only seven studies controlling for potential confounders.

All studies were included in the narrative synthesis, and all except four studies^11^, ^26^, ^29^, ^34^ were included in the meta-analysis (included participants n=31 212). These four studies were excluded from the meta-analysis because the exact number of voluntary or involuntary patients (or both) was not clearly stated. The full meta-analysis results are presented in table 2, and forest plots are provided in the appendix (pp 4–10).

**Table 2:** Risk factors for involuntary psychiatric hospitalisation based on meta-analysis of unadjusted data

		**Number of studies**	**Odds ratio (95% CI)**	**p value**	***I*^2^**
Intellectual disability (*vs* no intellectual disability)		4	3·33 (1·33–8·34)	0·010	65·6%
Primary diagnosis (*vs* those without the diagnosis)					
	Psychosis	8	3·63 (2·43–5·44)	<0·0001	90·5%
	Substance misuse	5	1·87 (1·05–3·30)	0·032	84·9%
	Behavioural disorder	6	0·71 (0·50–0·84)	0·0012	85·5%
	Anxiety disorder	2	0·19 (0·05–0·81)	0·025	0·0%
	Eating disorder	2	0·59 (0·03–11·87)	0·73	74·7%
	Mood disorder	6	1·02 (0·85–1·22)	0·84	66·7%
	Personality disorder	3	1·89 (0·35–9·93)	0·45	92·3%
	Developmental disorder	3	0·96 (0·49–1·87)	0·91	0·0%
Risk					
	Harm to self (*vs* no harm to self)	8	2·05 (1·15–3·64)	0·015	77·7%
	Harm to others (*vs* no harm to others)	5	2·37 (1·39–4·03)	0·0015	62·9%
Previous psychiatric admission (*vs* no previous admission)		3	2·18 (0·95–5·60)	0·10	77·8%
Female gender (*vs* male gender)		12	0·78 (0·55–1·11)	0·17	80·4%
Ethnicity (*vs* White)					
	Black	3	2·72 (1·88–3·95)	<0·0001	0·0%
	Asian	2	1·12 (0·32–3·84)	0·86	8·1%
	Other	2	1·21 (0·18–8·04)	0·85	62·1%
Age					
	Older adolescence (*vs* early adolescence)*	2	2·82 (1·04–7·63)	0·042	83·7%
	≥12 years (*vs* <12 years)	3	3·57 (1·46–8·73)	0·0052	90·4%
Living with family (*vs* not living with family)		4	0·40 (0·09–1·76)	0·23	74·9%
Previous abuse (*vs* none)					
	Any	2	1·07 (0·62–1·85)	0·80	0·0%
	Sexual	3	2·26 (0·88–5·82)	0·091	51·3%
	Physical	2	1·85 (0·51–6·76)	0·35	72·9%

* Older adolescence was defined as age 16–17 years and early adolescence as 12–15 years.

Our meta-analysis found that a diagnosis of intellectual disability was associated with involuntary rather than voluntary hospitalisation (four studies, OR 3·33, 95% CI 1·33–8·34, p=0·010). Intellectual disability was only clearly defined in one study, as an intelligence quotient of less than 80,^22^ and was given in addition to the primary diagnosis in all except one study.^27^

The odds of an involuntary rather than voluntary hospitalisation were higher for young people with a diagnosis of psychosis than for those without psychosis (eight studies; OR 3·63, 95% CI 2·43–5·44, p<0·0001). Young people with a primary (not comorbid) diagnosis of substance misuse were more likely to be hospitalised involuntarily than voluntarily (five studies; 1·87, 1·05–3·30, p=0·032). A diagnosis of behavioural problems (which included diagnoses such as attention-deficit hyperactivity disorder and conduct disorder) was associated with decreased odds of an involuntary rather than voluntary hospitalisation (six studies; 0·71, 0·50–0·84, p=0·0012), as was a diagnosis of anxiety disorder (two studies; 0·19, 0·05–0·81, p=0·025).

Young people who were perceived to be at risk of harm to themselves (including self-harm, suicidal ideation, or suicide attempts) had increased odds of an involuntary hospitalisation compared with those not at risk (eight studies; OR 2·05, 95% CI 1·15–3·64, p=0·015), as did those who were perceived to be at risk of harm to others (including aggression, violent acts, or danger to others; five studies; 2·37, 1·39–4·03, p=0·0015). Having had a previous psychiatric hospital admission was not associated with involuntary hospitalisation among children and adolescents.

With the exception of anxiety and developmental disorders, there was substantial heterogeneity identified for all of the clinical factors included in the meta-analysis (*I*^2^ from 66·7% to 92·3%). Because there are no clear outliers in terms of data, it is likely that this heterogeneity is due to the variety of methods used to make clinical decisions about diagnosis and risk, as well as characteristics of different health and legal systems. In addition, the analysis of most of these variables was based on a small number of studies.

In terms of sociodemographic factors, we did not identify any association between gender and involuntary hospitalisation, although heterogeneity was very high (*I*^2^=80·4%). Few studies considered ethnicity, and categorisation was often crude when it was included, with a lack of clarity as to whether it was self-reported. However, the data recorded showed that the odds of an involuntary rather than a voluntary hospitalisation among children and adolescents from Black ethnic groups (including Black British, Black Caribbean, Black African, African American, and Black Other) was higher than those for young people from White ethnic groups (White British, White Irish, or White Other; three studies; 2·72, 1·88–3·95, p<0·0001). Among young people from Asian ethnic groups (Indian, Pakistani, Bangladeshi, Asian, or Other) and other ethnic groups (an ethnic group not listed or mixed ethnic origin), there was no significant difference in the risk of involuntary versus voluntary hospitalisation compared with young people from White groups, although this analysis was based on only two studies. For the analyses of ethnicity statistical heterogeneity was low.

Four studies (three from the UK and one from the USA) examined the association between ethnicity and involuntary hospitalisation further. In a UK-based historical cohort study, Corrigall and Bhugra^24^ found that differences in the use of the Mental Health Act according to ethnicity only occurred in those with psychosis. Young people from Black and Other ethnic groups with psychosis were more likely to be detained under the Mental Health Act at any point in their admission than those with psychosis in the White group (OR 3·0, 95% CI 1·3–6·7 for Black participants and 3·1, 1·1–8·8 for participants of Other ethnicities). In the non-psychosis group, there were no significant differences in use of the Mental Health Act.^24^ Kilgus and colleagues^30^ found that during a 1-year period in a state hospital facility in South Carolina, African American adolescents were twice as likely to be involuntarily hospitalised at the time of admission than White American adolescents (OR 2·051, p=0·043), controlling for both gender and diagnosis.^30^ In a UK cross-sectional study, Tolmac and Hodes^41^ found that young Black people were significantly more likely to be detained under the Mental Health Act than young White people on admission. However, when looking at the use of the Mental Health Act at any point during the hospitalisation, there was no significant difference between the ethnic groups.^41^

Older adolescents (16–17 years old) were more likely to be involuntarily hospitalised than those aged 12–15 years (two studies, OR 2·82, 95% CI 1·04–7·63, p=0·042). In addition, adolescents aged 12 years or older were more likely to have an involuntarily rather than voluntary admission compared with those younger than 12 years (three studies; 3·57, 1·46–8·73, p=0·0052).

We found no evidence of an association between involuntary hospitalisation and whether a young person was living with their parents or family at the time of admission, although none of the four relevant studies clearly specified the living arrangements of those not living with family, so these participants could have included those living with friends, in an institution, or in foster care. Having a previous history of experiencing any type of abuse, physical abuse, or sexual abuse was not associated with involuntary hospitalisation. Although the data were not suitable for meta-analysis, Ottisova and colleagues^34^ found, contrary to their hypothesis, that young victims of trafficking (74% of whom had been subjected to physical or sexual violence) were no more likely to be involuntarily rather than voluntarily admitted for psychiatric inpatient care than those who had not been trafficked, despite the high rate of self-harm (33%) and suicide attempts (27%) identified in the trafficked group.

Among included studies, there was no evidence of publication bias through visual examination of the funnel plots (appendix pp 16–24). We were able to do one meta-regression on publication year (before 2010 *vs* 2010 or later), but further post-hoc analysis was not possible due to the small number of studies. Restricting the analysis to high-quality studies was not feasible for the same reason. The meta-regression on publication year identified that in studies published in 2010 or later, young people with personality disorder were more likely to be admitted voluntarily than involuntarily (appendix p 15). There was no evidence that publication date was associated with the legal status of admission for any of the other variables.

The narrative synthesis included all 23 studies. Most studies measured differences in gender between the voluntary and involuntary patients, but only four studies stratified the legal and diagnostic groups by gender. Jendreyschak and colleagues^27^ found that in those younger than 12 years, having a diagnosis of psychosis or intellectual disability and being male was significantly associated with an involuntary rather than voluntary admission. In patients aged 12 years or older, both male and female patients with a diagnosis of substance misuse disorders, psychosis, neurotic disorders, or intellectual disability were significantly more likely to be admitted involuntarily than voluntarily (the study was rated as high quality).^27^ Mears and colleagues^33^ found that most of the involuntary patients with mood disorder diagnoses were female and most of those admitted involuntarily with a diagnosis of schizophrenia were male (the study was rated as low quality). In a high-quality Finnish register study, Kaltiala-Heino^10^ found that affective and neurotic disorders were the most common diagnoses among the female patients who were admitted involuntarily, whereas conduct disorders, psychotic disorders, and substance misuse were the most common diagnoses in the male patients who were admitted involuntarily. In a later, smaller, but also high-quality study (n=187), Kaltiala-Heino^28^ found that hostility, “temper tantrums”, or breaking property were significantly associated with being referred to hospital involuntarily, but only in girls.

A range of measures was used to record the young people's psychiatric symptoms and level of functioning. These included the Children's Global Assessment Scale, Health of the Nation Outcome Scales for Children and Adolescents, Beck Depression Inventory-II, Brief Psychiatric Rating Scale, Global Assessment of Functioning, State-Trait Anxiety Inventory, and the Child Behaviour Checklist. In the seven studies in which these rating scales were used, five studies (three rated high quality, one moderate quality, and one low quality) found that young people admitted involuntarily had scores indicative of substantially more severe clinical presentation or poorer levels of functioning than those hospitalised voluntarily.^22^, ^25^, ^31^, ^32^, ^38^ These findings could not be included in the meta-analysis due to variation in how results were reported.

Mears and colleagues^33^ used the Health of the Nation Outcome Scales for Children and Adolescents, but instead of giving overall scores, they detailed the results of the individual sections. They found that those admitted involuntarily to 71 inpatient units in England and Wales had significantly more hallucinations and delusions, peer relationship problems, and family problems than those admitted voluntarily. However, those with physical illnesses, somatic symptoms, and emotional difficulties were significantly more likely to be admitted voluntarily than involuntarily.^33^ A moderate-quality Canadian study by Persi and colleagues^36^ found that there was no difference in clinical presentation or global level of functioning between the voluntary and involuntary patients, with no significant differences between the Children's Global Assessment Scale or the Child Behaviour Checklist scores between the two groups. However, although 80% of the patients were admitted involuntarily, only 11% of the patients remained involuntarily detained after psychiatric review, leading the authors to suggest that involuntary admissions might be overused.

Only two studies reported whether any previous psychiatric hospital admissions were involuntary.^29^, ^38^ Khenissi and colleagues^29^ found that more of the involuntary than voluntary patients had previously been sent for involuntary treatment (51·3% *vs* 14·9%, p<0·001). However this study was not included in our meta-analysis because the precise number of voluntary patients was not stated. A high-quality study by So and colleagues^38^ also identified that a previous involuntary admission was significantly associated with involuntary versus voluntary admission (p<0·01).

Contact with community psychiatric services before admission was reported in two high-quality studies.^28^, ^38^ So and colleagues^38^ found that a lack of medical compliance and a lack of motivation for treatment, measured on the Severity of Psychiatric Illness Scale, were both significantly associated with involuntary hospitalisation on multivariate analysis, although it is unclear whether these scores relate to previous levels of motivation and medical compliance, or compliance with the emergency assessment during which the scale was administered. Kaltiala-Heino^28^ identified that the young people hospitalised involuntarily were significantly more likely to have been referred to the psychiatric hospital by primary care or non-psychiatric specialists, whereas those who were admitted voluntarily were more likely to have been referred by an adolescent psychiatrist. In the study by So and colleagues,^38^ these findings were reversed, and the young people admitted involuntarily were more likely to have been referred by psychiatric services than by a general practitioner.

Involvement of social care is mentioned in three studies.^25^, ^26^, ^31^ Ellila and colleagues^25^ found that a planned out-of-home placement on discharge from hospital was associated with involuntary treatment. Jaworowski and Zabow^26^ found that most of the involuntary patients in their study were referred by social services but no further detail is given. Only one study specifically included data on whether young people were adopted or in a foster placement before the hospital admission and found that these young people were significantly more likely to be admitted involuntarily than voluntarily.^31^

One longitudinal Finnish study compared voluntary and involuntary hospitalisations across districts and identified that involuntary hospitalisations of children and adolescents increased substantially from 1996 to 2003.^11^ The authors suggest that this increase could be due to the economic recession, which might have limited the availability of outpatient resources. Additionally, they identified that in areas with high rates of involuntary hospitalisation, child welfare placements were considerably more common. The reason for this finding is not clear, but the authors suggest that it could be related to regional differences in the resources available to support young people effectively in the community. None of the other studies included in the review considered the potential association between socioeconomic status and involuntary care.

Six studies included multivariate analyses, adjusting for factors potentially associated with involuntary hospitalisation. Ellila and colleagues^25^ identified seven factors which were significantly associated with involuntary hospitalisation on univariate regression analysis: substance use disorder, suicidal act, psychosis, violent act, out-of-home placement, Children's Global Assessment Scale score of less than 40, and age 16–17 years. When all of these factors were controlled for, only three (substance use disorder, suicidal act, and psychosis) were independently associated with involuntary legal status. They also found that there was no significant gender–age interaction.^25^ Jendreyschak and colleagues^27^ used direct logistic regression to assess the effect of ten variables on the likelihood of being admitted to hospital involuntarily. Of these, seven made a highly significant contribution (p<0·001): age 12–17 years, substance use, psychotic disorder, intellectual disability, behavioural disorders, anxiety disorders, and being admitted in duty time (recorded as 1600 h to 0800 h). Three other factors made a significant contribution (p<0·01): male gender, affective disorder, and previous admission. The strongest predictor for involuntary hospitalisation was having intellectual disability (OR 15·74, 95% CI 10·82–22·90).^27^ Sourander and colleagues^39^ also found that a diagnosis of psychosis and older age (15–17 years *vs* 12–14 years) were significantly associated with involuntary hospitalisation on multivariate analysis, controlling for gender, whether or not it was a first admission, whether they were admitted to an adult or adolescent unit, and the treatment year. On stepwise multiple logistic regression analysis, So and colleagues^38^ found that any DSM-IV Axis 1 diagnosis, high risk of suicide, danger to others, previous compulsory care, and lack of motivation or compliance all predicted involuntary rather than voluntary hospitalisation.

## Discussion

Despite the paucity of literature on this topic, our systematic review, meta-analysis, and narrative synthesis have identified a number of clinical and social factors that are associated with an increased likelihood of involuntary over voluntary psychiatric hospitalisation in children and adolescents. The clinical factors include a diagnosis of psychosis, substance misuse, or intellectual disability, as well as the presence of perceived risk of harm to self or others. On narrative synthesis, more severe psychiatric symptoms and poorer levels of functioning also seem to be related to involuntary rather than voluntary hospitalisation. Anxiety and behavioural disorders were associated with voluntary rather than involuntary hospitalisation. In terms of sociodemographic factors, older age and being from a Black rather than a White ethnic group were associated with involuntary rather than voluntary hospitalisation.

The over-representation of adults from Black and minority ethnic groups in hospital involuntarily in the UK and globally has long been recognised and is associated with structural and institutional factors that lead to the systematic disadvantage of people from minority ethnic groups.^7^, ^42^, ^43^, ^44^, ^45^ However, little attention has been given to the role that structural racism plays in the mental health care of children and adolescents, and the effect on health outcomes of early experiences of discrimination.^46^ We were only able to identify seven studies that mentioned the ethnicity of the children and adolescents who were involuntarily detained,^24^, ^30^, ^32^, ^35^, ^36^, ^37^, ^41^ compared with 71 studies included in a recent international meta-analysis of ethnic variations in involuntary hospitalisation among adults.^7^

Research in adults from the UK has consistently found that people from minority ethnic groups are more likely to be diagnosed with severe mental illness than White people.^45^ However, a national survey in 2017 of mental health in children and adolescents in England found that children and adolescents from Black and minority ethnic backgrounds were less likely than those from White ethnic backgrounds to have any mental disorder.^47^ It is essential that we understand more about the reasons behind the diagnostic discrepancies between different ethnicity and age groups. A number of studies have identified that, as with adults,^48^, ^49^ young people from Black and minority ethnic groups are more likely than young people from White ethnic groups to be referred to mental health services via the criminal justice system or social care, rather than through less coercive routes, such as a family doctor.^50^, ^51^, ^52^, ^53^ Socioeconomic factors might play a role in these adverse pathways, but even within similar socioeconomic statuses, the additional racism and discrimination experienced by those from minority ethnic groups is associated with worse health outcomes, particularly in children and adolescents.^54^, ^55^ It is unfair that the existence and potential causes of ethnic inequalities in involuntary hospitalisation among children and adolescents has received so little academic, clinical, and political attention to date, and a systematic assessment of the role of race in involuntary hospitalisation of young people should be a focus of urgent further investigation.^56^

The association between involuntary psychiatric hospitalisation and intellectual disability is also concerning, although these findings are based on a small number of studies, and it is unclear from the data provided which comorbid psychiatric disorders, if any, the young people with intellectual disability had, and what specifically precipitated the admission. In addition, intellectual disability is an extremely heterogeneous diagnosis and only one study provided a definition. In the UK, a recent joint House of Commons and House of Lords report notes that that, “when young people [with an intellectual disability] are detained it is usually the result of a long and predictable series of failures to appropriately support them and their family”.^57^ Involuntary psychiatric hospitalisation is in itself a poor outcome and the increased odds of involuntary over voluntary psychiatric hospitalisation among young people with intellectual disabilities identified in this review highlights the need for urgent further investigation into the systemic failures to provide appropriate and timely care for these young people and their families.

The strong association between a diagnosis of psychosis and involuntary hospitalisation corresponds with the adult literature.^6^ However, the specific factors that predict whether a young person with psychosis is hospitalised involuntarily or voluntarily remain unclear. A UK study found that young Black people with psychosis were more likely to have an involuntary hospitalisation than young White people with psychosis,^24^ which implies that this decision could somehow be influenced by racial biases. This issue requires urgent further investigation.

A primary diagnosis of substance misuse disorder was the only other diagnosis that we found to be associated with involuntary rather than voluntary hospitalisation among children and adolescents. There is evidence in adult populations to suggest that substance misuse cannot be treated coercively,^58^ and some evidence that outpatient treatment with family therapy is the most effective treatment for young people who misuse substances.^59^ Given the potentially poor outcomes of substance misuse in young people and the effects on the developing brain, we must seek to understand more about these involuntary admissions and improve the availability of community interventions, which are increasingly hard to access (at least in the UK) due to reductions in funding for adolescent substance misuse services.^60^, ^61^

Young people presenting as a perceived risk to themselves or others are significantly more likely to be hospitalised involuntarily than voluntarily. This is perhaps to be expected given that risk is one of the criteria for detention in most mental health legislation internationally.^13^ However, in adults, risk to others is associated with involuntary admission, whereas risk to self is more likely to be associated with a voluntary admission.^6^ The reasons for these differences are likely to be multifaceted and could include differences in the way young people present with self-harm or suicidal ideation; fears of increased impulsivity among adolescents; an increased sense of responsibility to protect the young; and differences in the availability of alternative support networks. The association of behavioural disorders such as attention-deficit hyperactivity disorder and conduct disorder with voluntary rather than involuntary hospitalisation is perhaps surprising given that these diagnoses can also be associated with aggressive behaviour and impulsivity.^62^ Personality disorder was also associated with voluntary rather than involuntary hospitalisation, but only in the studies from 2010 or later. This is interesting from a UK perspective because one of the amendments to the Mental Health Act in 2007 was designed to make it easier to involuntarily admit people with personality disorder who were thought to be at high risk.^63^ However, only one of the studies that included data on personality disorder in this review came from the UK, and this study was published before 2010. The studies included in this review do not describe how risk is assessed and reported in children and adolescents and how this might influence decisions about the legal status of admission. This should be an important focus of future research in this field. It is also important to consider family functioning and parental capacity to support a young person with psychosis, substance misuse, intellectual disability, or risk in the community, and understand how this could affect the likelihood of an involuntary over a voluntary (possibly under parental consent) hospitalisation.

In the adult population, men are more likely to be hospitalised involuntarily than women, but our study found no association between gender and the legal status of hospital admission in children and adolescents. Further research is needed to understand the potential influence of gender on risk of involuntary hospitalisation among young people, including whether gendered perceptions of risk and expectations of behaviour could influence clinical decision making about detention. One Finnish study identified that hostile behaviour, “temper tantrums”, and breaking property was associated with involuntary referral to hospital, but only in girls.^28^ A possible explanation for this finding is that boys who demonstrate antisocial behaviour might be more likely than girls to be diverted to the criminal justice system,^64^ but research in this area is scarce and it is not discussed in any of the studies in this review.

Older age was also strongly associated with involuntary hospitalisation. This finding fits with the onset in later adolescence of the more severe mental disorders, such as psychosis and substance misuse. In addition, younger people can sometimes be admitted to hospital voluntarily under parental consent, but this becomes more problematic as the young person increases in age. The age at which a young person can be admitted under parental consent varies between countries (eg, younger than 16 years in the UK and younger than 13 years in France^31^) but detailed information about international variations in the application of mental health legislation among children and adolescents is not available, and would be a useful avenue for further research.^65^

In the studies in this review, little information was available on looked-after young people (those in the care of social services), who are vulnerable to mental health difficulties and adverse outcomes.^66^ There was also little information on pathways into involuntary care, including police involvement, or previous involuntary hospitalisation. None of the studies measured the socioeconomic status of the young inpatients, despite the known associations between poverty and poor mental health outcomes. Measures of socioeconomic disadvantage on both individual and population levels must be included in further research in this field to enable an understanding of how socioeconomic factors interact with the other variables we have identified and affect involuntary hospitalisation among children and adolescents.

Our study has several limitations. As an international review, we have included studies from a range of countries with different legal criteria for involuntary hospitalisation, and different mental health systems and processes. This, along with a range of study methods, settings, and time periods, has probably contributed to the high heterogeneity between studies. The substantial heterogeneity and small number of studies mean that the pooled data need to be interpreted with caution. The studies are all from high-income countries, which precludes any investigation into the involuntary hospitalisation of children and adolescents in middle-income and low-income countries, where specialist child services and expertise can be rare. Future research in this field would benefit from the inclusion of a wider range of sources. These could include qualitative studies of the experiences of young people and carers in involuntary hospitalisation settings and the circumstances that preceded it; clinician views on the decision-making processes around involuntary care; and the use of primary data sources, including routine databases and linked clinical, social, and police records. We have focused on young people detained in hospital under mental health legislation, but some young people are admitted to hospital voluntarily under parental or guardian consent, which is a de facto involuntary admission. It would be helpful to know more about the differences between these types of admissions in terms of risk factors, experiences, and outcomes, and this should be the focus of future research. However, the main limitation is the paucity of research into the involuntary hospitalisation of young people such that our meta-analysis was limited for some variables to only two studies. The small number of studies meant that further exploration of potential confounders through meta-regression and sensitivity analysis was not possible (with the exception of publication year). Although we were able to identify several variables associated with involuntary rather than voluntary hospitalisation of children and adolescents, and our findings were supported by the results of the multivariate analyses done in six of the studies, our understanding of the interactions between the variables and their mechanisms of influence remains poor. The data available also precluded any analysis of the effects of other potentially important factors, such as the role of parents.

Despite these limitations our study is, to our knowledge, the first international review of the social and clinical factors associated with the involuntary hospitalisation of children and adolescents. For adults, engagement in crisis services and advance directives can help reduce the rate of involuntary hospitalisations,^67^, ^68^, ^69^, ^70^ but further research is needed to understand which interventions would work best for young people, with specific consideration of socioeconomic, racial, and ethnic factors. This work needs to be done alongside prospective research into the factors associated with involuntary hospitalisation among children and adolescents, and how these might change as the young people become adults; as well as qualitative research into the experiences of involuntary hospitalisation for children, adolescents, their parents or guardians, and the professionals who make the decisions to detain. A lived-experience commentary on this study is provided in the panel.

Panel **Interpretation of findings by lived-experience panellists Jummy Otaiku and Patrick Nyikavaranda**

In this paper by Walker and colleagues, it is of interest that clinical and social factors appear to be significantly associated with involuntary psychiatric hospitalisation of young people. The report raises concerns about systemic bias during the decision-making process. Being from a Black, Asian, or minority ethnic background or having an intellectual disability, a young person is more likely to be involuntarily than voluntarily detained, not necessarily because of the need for mental health care, but because of their characteristics. Similar to the adult population, young people from minority groups may first encounter treatment for a mental health condition through the criminal justice system. There does not appear to be much evidence about what could be done to prevent involuntary hospitalisations before they arise. As the paper describes, intervening early has the potential to alter an individual's mental health trajectory, and it could prevent future hospitalisation for a lot of cases. With the rise in involuntary hospitalisation across the UK, and indeed, worldwide, there appears an urgent need to investigate the identified factors further, with a particular push to involve young people and their carers in the design of preventive interventions. Walker and colleagues acknowledge the benefits of hospitalisation but also state that it can be a traumatic experience. It is not known how many young people do get voluntarily hospitalised when they are sometimes viewed as not having the capacity or the power to object without fear of coercive means. The studies in this paper were from high-income countries, and few considered ethnicity. Despite an increase in the global movement of people, there is a lack of evidence of clinical and social factors of migrant and looked-after children, an area that is yet to be explored concerning involuntary hospitalisation.

We hope that a greater understanding of the factors associated with involuntary psychiatric hospitalisation of children and adolescents will contribute to the creation of more equitable pathways to psychiatric treatment for patients of all ages and, ultimately, a reduction in long-standing health-care inequalities.

## Data sharing

All data collected for this Article, including data extraction tables and the statistical analysis, will be available from the publication date. Requests to access these data should be made to the corresponding author.

## Declaration of interests

We declare no competing interests.
