# Supplementary material for: Clinical and social factors associated with involuntary psychiatric hospitalisation in children and adolescents: a systematic review, meta-analysis, and narrative synthesis
Source: Lancet Child Adolesc Health. 2021 Jul;5(7):501–12. doi: 10.1016/S2352-4642(21)00089-4 (PMC8205858; doi:10.1016/S2352-4642(21)00089-4)

THE LANCET
Child & Adolescent Health

Supplementary appendix

This appendix formed part of the original submission and has been peer reviewed. We post it as supplied by the authors.

Supplement to: Walker S, Barnett P, Srinivasan R, Abrol E, Johnson S. Clinical and social factors associated with involuntary psychiatric hospitalisation in children and adolescents: a systematic review, meta-analysis, and narrative synthesis. *Lancet Child Adolesc Health* 2021; published online April 27. https://doi.org/10.1016/S2352-4642(21)00089-4.

Appendix

Search Terms

Medline

1. exp Mental Health Services/
2. exp Emergency Services, Psychiatric/
3. Hospitals, psychiatric/ or Psychiatry Department, Hospital.mp. [mp=title, abstract, original title, name of substance word, subject heading word, keyword heading word, protocol supplementary concept word, rare disease supplementary concept word, unique identifier, synonyms]
4. (psychiatr* adj3 (admission* or admitt* or readmi* or re-admi* or hospitali* or in-patients or inpatients)).ti,ab,kf.
5. Mentally Ill Persons.mp. or exp Mentally Ill Persons/
6. mental health/ or mental disorders/
7. ((mental or psychiatr*) adj (health or disorder* or disease* or deficien* or illness* or problem*)).mp. [mp=title, abstract, original title, name of substance word, subject heading word, keyword heading word, protocol supplementary concept word, rare disease supplementary concept word, unique identifier, synonyms]
8. or/1-7
9. "Commitment of Mentally Ill".mp. or exp "Commitment of Mentally Ill"/
10. Involuntary Treatment.mp. or exp Involuntary Treatment/
11. commitment.ti.
12. ((psychiatr* or mental* or psychos* or schizo*) adj3 commit*).ti,ab,kf.
13. ((commitment or restriction or court) adj2 order?).ti,ab,kf.
14. ((mental health adj (act? or jurisdiction or law? or legal* or legislat*)) and (admission* or admitt* or readmi* or re-admi* or hospitali* or in-patients or inpatients or commit* or detain* or detention* or section* or treat* or care)).mp.
15. ((compulsory or forced or involunt* or in-volunt* or mandat*) adj3 (admission* or admitt* or readmi* or re-admi* or hospitali* or in-patients or inpatients or commit* or detain* or detention* or section* or treat* or care)).ti,ab,kf.
16. or/9-15
17. child*.mp. or exp CHILD/
18. exp Adolescent/ or adolescen*.mp.
19. teen*.mp.
20. exp Infant/ or infan*.mp. or exp Child, Preschool/
21. or/17-20
22. 8 and 16 and 21

Appendix

Psychinfo

1. exp "Commitment (Psychiatric)"/ or "Commitment (Psychiatric)".mp.
2. Involuntary Treatment/
3. ((psychiatr* or mental* or psychos* or schizo*) adj3 commit*).ti,ab,id.
4. ((mental health adj (act? or jurisdiction or law? or legal* or legislat*)) and (admission* or admitt* or readmi* or re-admi* or hospitali* or in-patients or inpatients or commit* or detain* or detention* or section* or treat* or care)).ti,ab,id.
5. ((compulsory or forced or involunt* or in-volunt* or mandat*) adj3 (admission* or admitt* or readmi* or re-admi* or hospitali* or in-patients or inpatients or commit* or detain* or detention* or section* or treat* or care)).ti,id.
6. ((commitment or restriction or court) adj2 order?).ti,ab,id.
7. legal detention/
8. or/1-7
9. child*.mp.
10. adolescen*.mp.
11. teen*.mp.
12. infan*.mp.
13. or/9-12
14. 8 and 13

Appendix

Embase

1. Mental Health Service/
2. Psychiatric Emergency service/
3. Mental Hospital/ or Mental Patient/
4. Psychiatric Department/
5. (psychiatr* adj3 (admission* or admitt* or readmi* or re-admi* or hospitali* or in-patients or inpatients)).ti,ab,kw.
6. psychiatric.ti,kw,hw. and Hospital Patient/
7. ((mental or psychiatr*) adj (health or disorder* or disease* or deficien* or illness* or problem*)).ti,ab,kw.
8. Mental Health/ or Mental Disease/
9. or/1-8
10. involuntary commitment/
11. commitment.ti.
12. ((psychiatr* or mental* or psychos* or schizo*) adj3 commit*).ti,ab,kw.
13. ((commitment or restriction or court) adj2 order?).ti,ab,kw.
14. ((mental health adj (act? or jurisdiction or law? or legal* or legislat*)) and (admission* or admitt* or readmi* or re-admi* or hospitali* or in-patients or inpatients or commit* or detain* or detention* or section* or treat* or care)).mp.
15. ((compulsory or forced or involunt* or in-volunt* or mandat*) adj3 (admission* or admitt* or readmi* or re-admi* or hospitali* or in-patients or inpatients or commit* or detain* or detention* or section* or treat* or care)).ti,ab,kw.
16. or/10-15
17. child*.mp.
18. infan*.mp.
19. adolescen*.mp.
20. exp adolescence/
21. exp childhood/
22. exp infancy/
23. or/17-22
24. 9 and 16 and 23

Appendix

Forest Plots

Involuntary hospitalisation of women vs men

Author	Women: N detained	Women: Total N	Men: N detained	Men: Total N	Odds ratio [95% CI]
Ayton 2009	16	47	0	3	3.67 [0.18, 75.32]
Ellila 2008	48	152	34	126	1.25 [0.74, 2.10]
Jendreyschak 2013	1423	4506	1658	6041	1.22 [1.12, 1.33]
Laget 2002	13	46	3	20	2.23 [0.56, 8.92]
Lindsey 2010	142	178	158	205	1.17 [0.72, 1.91]
Mears 2003	61	381	64	278	0.64 [0.43, 0.94]
Mertons 2017	5	14	7	10	0.24 [0.04, 1.36]
Park 2011	83	169	121	163	0.33 [0.21, 0.53]
Persi 2016	107	136	73	89	0.81 [0.41, 1.59]
Ramel 2015	8	149	20	112	0.26 [0.11, 0.62]
So 2019	57	143	33	84	1.02 [0.59, 1.78]
Stein 1988	10	23	13	23	0.59 [0.18, 1.90]
RE Model for women (I^2 = 80.35%)					0.78 [0.55, 1.11]

Risk of involuntary hospitalisation in women

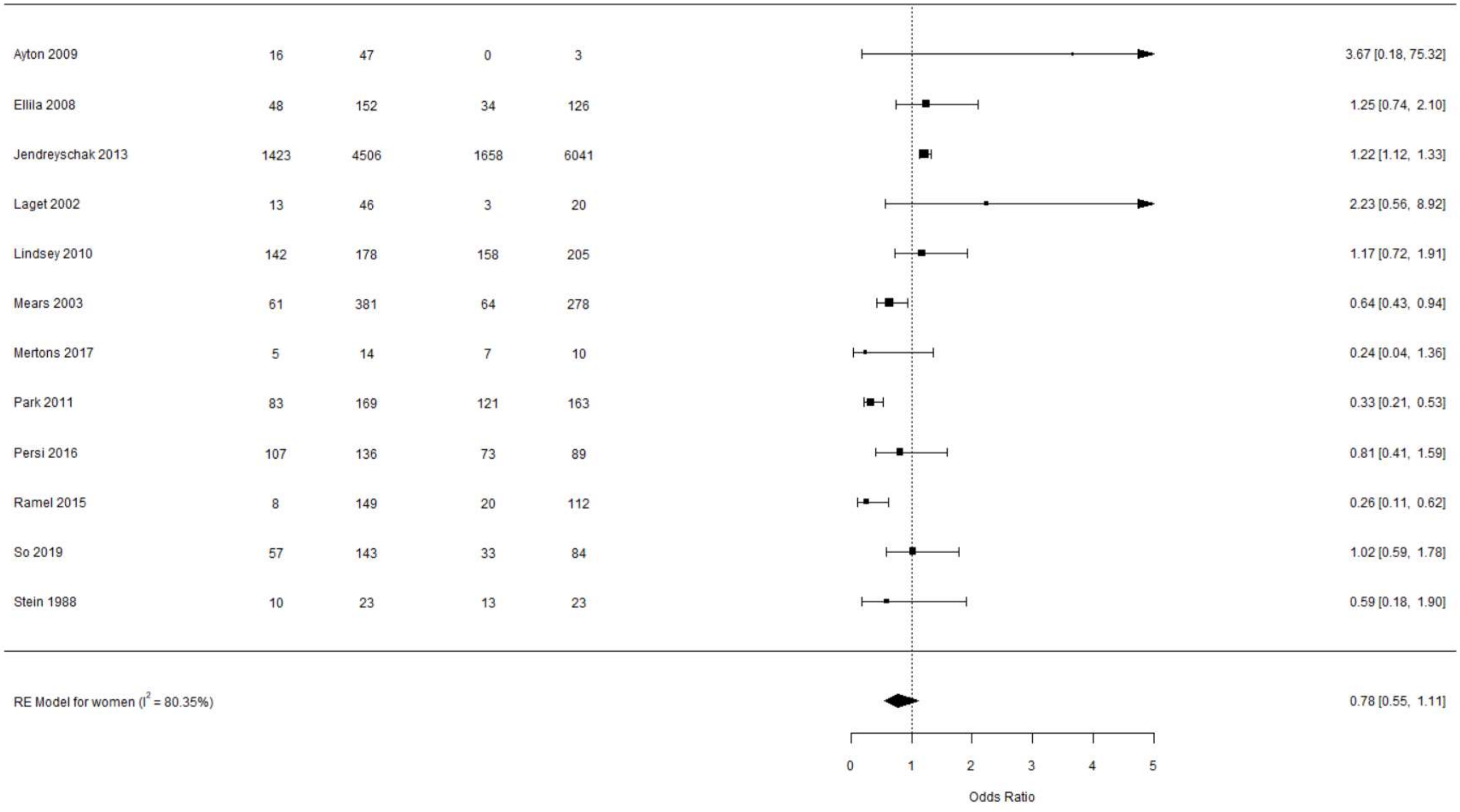

Appendix

Risk to others

Author	Aggression: N detained	Aggression: Total N	No aggression: N detained	No aggression: Total N	Harm to others	Odds ratio [95% CI]
Ellila 2008	25	50	57	228		3.00 [1.60, 5.63]
Kaltiala-Heino 2010	15	53	27	134		1.56 [0.75, 3.25]
Mears 2003	32	135	95	528		1.42 [0.90, 2.23]
Mertons 2017	10	17	2	6		2.86 [0.41, 20.14]
So 2019	34	49	56	178		4.94 [2.49, 9.80]
RE Model (I^2 = 62.90%)						2.37 [1.39, 4.02]

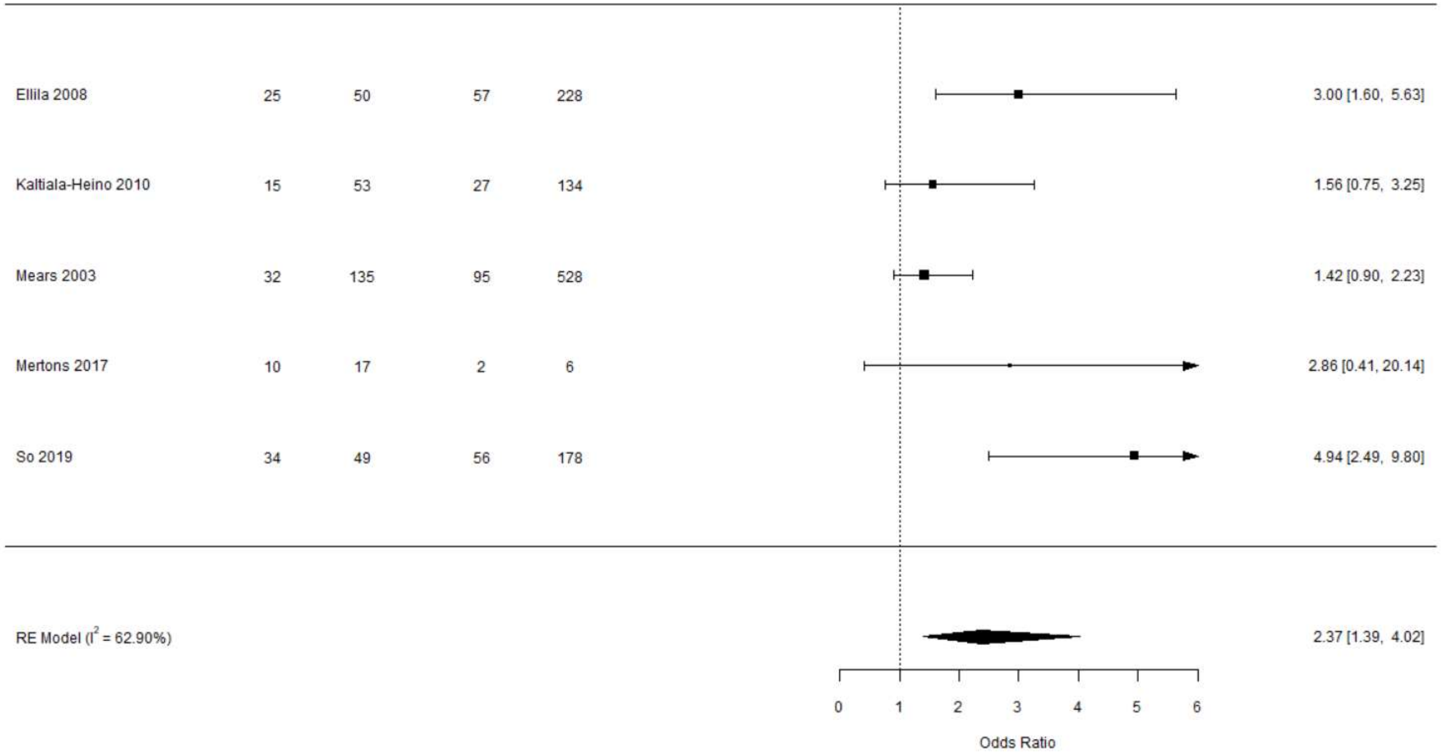

Appendix

Risk to self

Author	Self Harm N detained	Self Harm Total N	No Self Harm N detained	No Self Harm Total N	Odds ratio [95% CI]
Ayton 2009	12	16	4	34	22.50 [4.83, 104.86]
Ellila 2008	25	43	57	235	4.34 [2.21, 8.52]
Kaltiala-Heino 2010	15	75	27	112	0.79 [0.39, 1.60]
Lindsey 2010	61	74	239	309	1.37 [0.71, 2.65]
Mears 2003	18	85	109	578	1.16 [0.66, 2.02]
Mertons 2017	4	10	8	13	0.42 [0.08, 2.25]
Persi 2016	160	192	20	33	3.25 [1.47, 7.20]
So 2019	62	126	28	101	2.53 [1.45, 4.41]
RE Model (I^2 = 77.66%)					2.04 [1.15, 3.64]

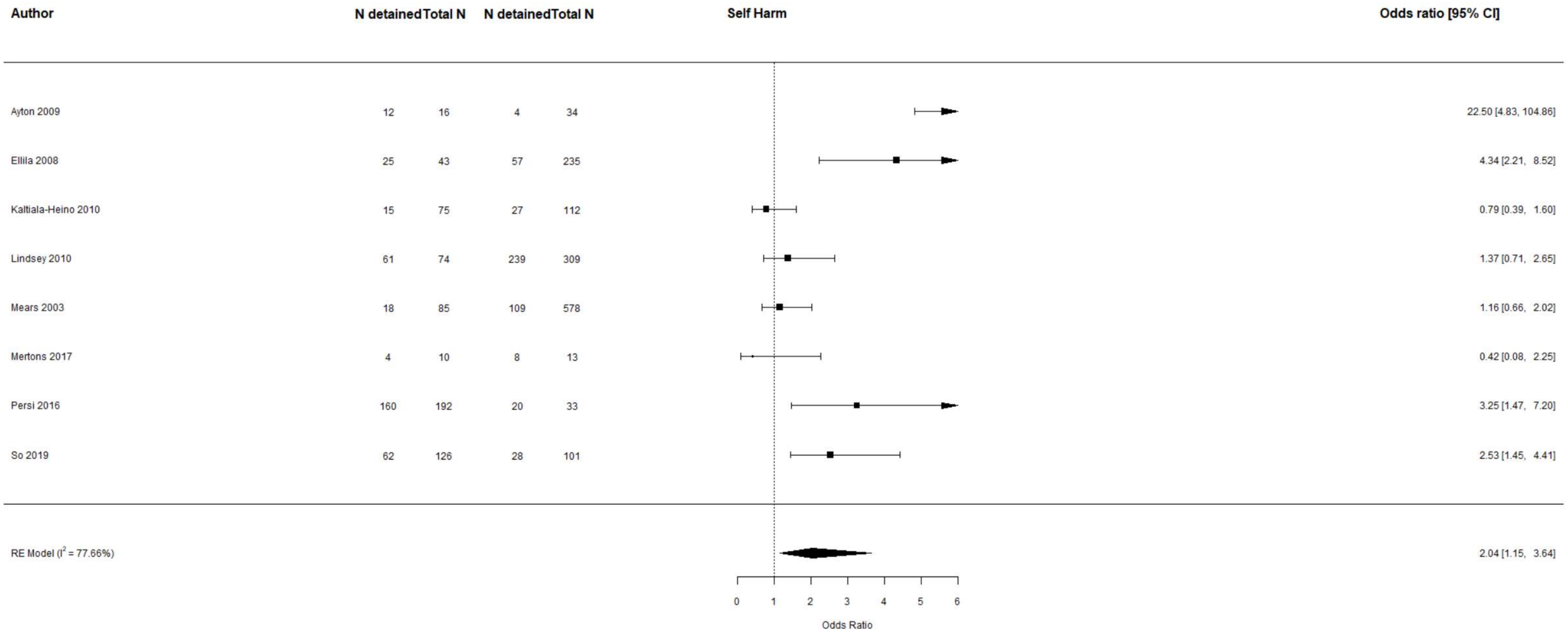

Appendix

Previous abuse

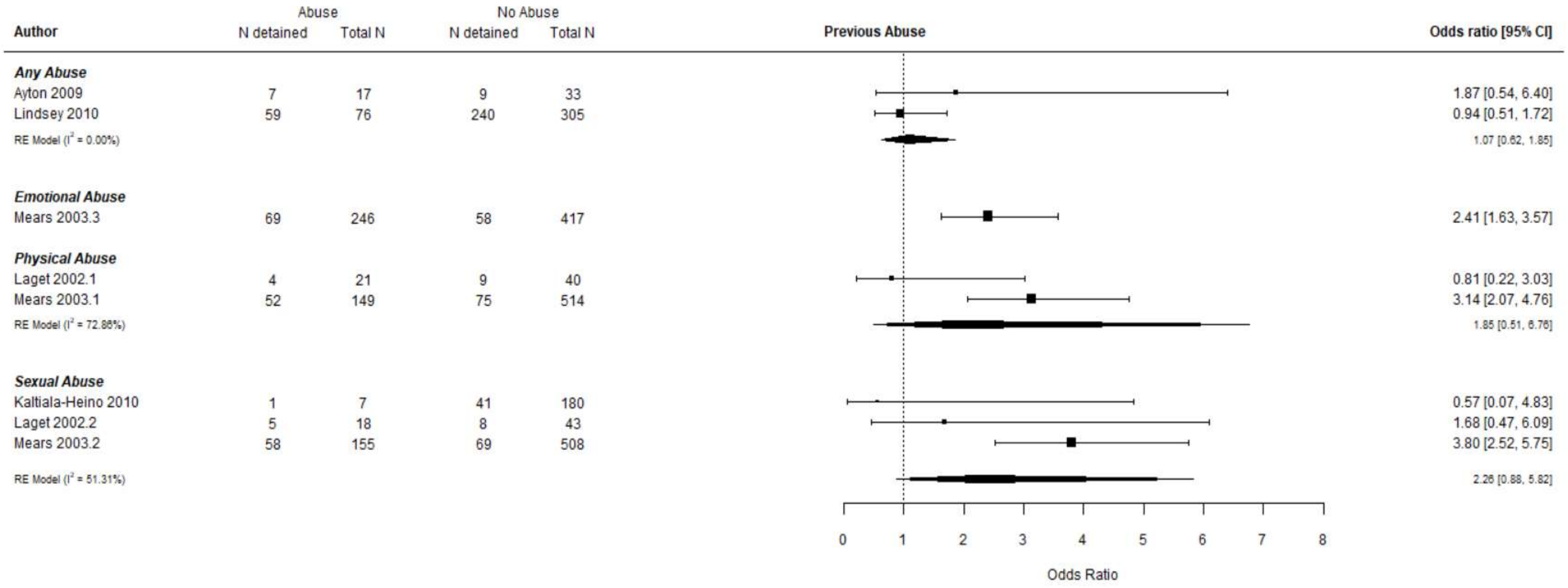

Appendix

Ethnicity

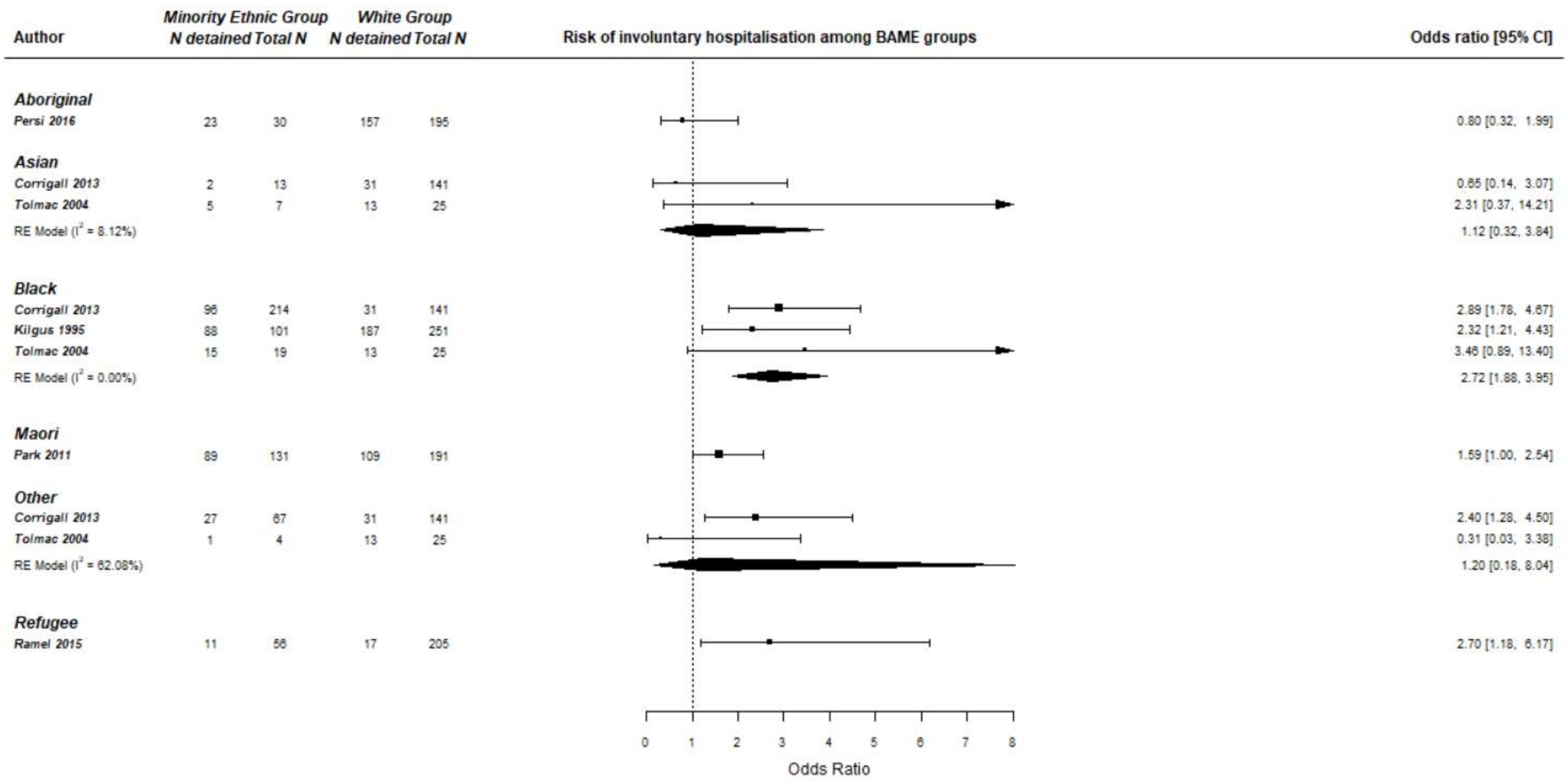

Appendix

Living situation

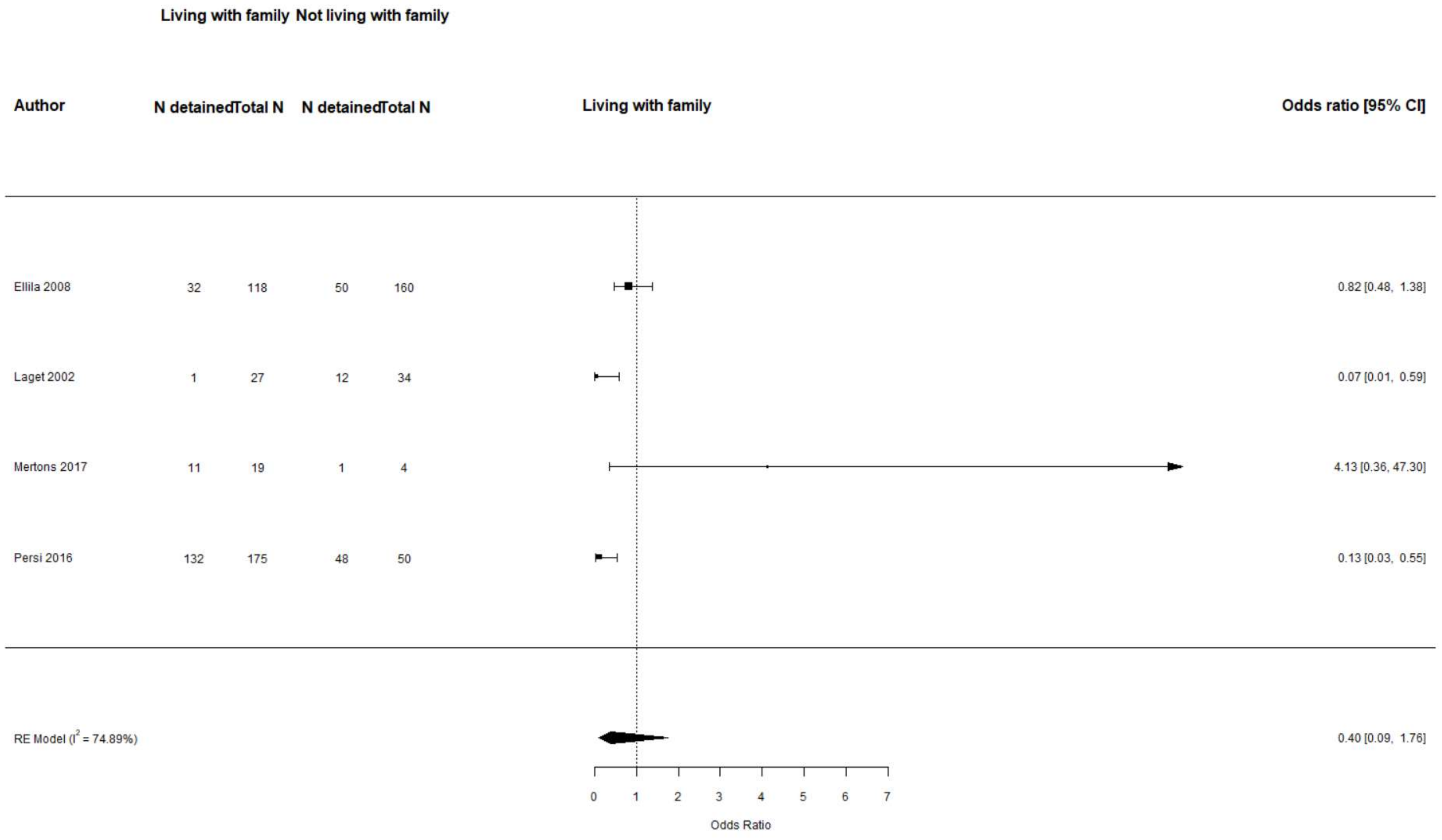

Appendix

Previous admission

	Previous admission		No Previous Admission			
Author	N detained	Total N	N detained	Total N	Previous Admission	Odds ratio [95% CI]
Ayton 2009	14	24	2	26		16.80 [3.21, 87.92]
Ellila 2008	31	98	51	180		1.17 [0.69, 2.00]
So 2019	32	68	58	159		1.55 [0.87, 2.75]
RE Model (I^2 = 77.82%)						2.18 [0.85, 5.56]

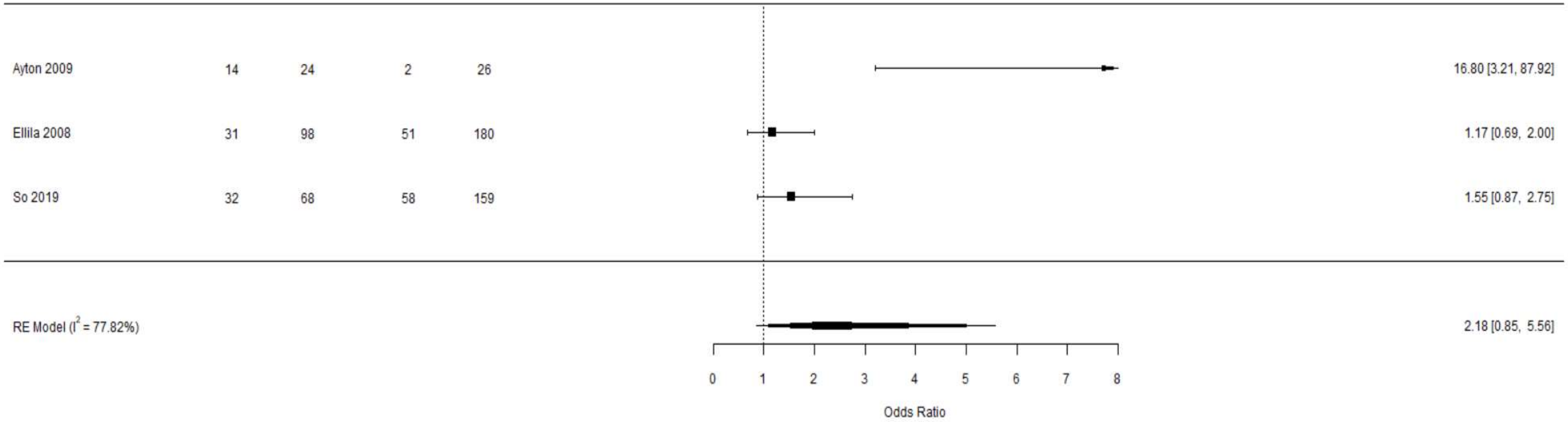

Appendix

Diagnosis

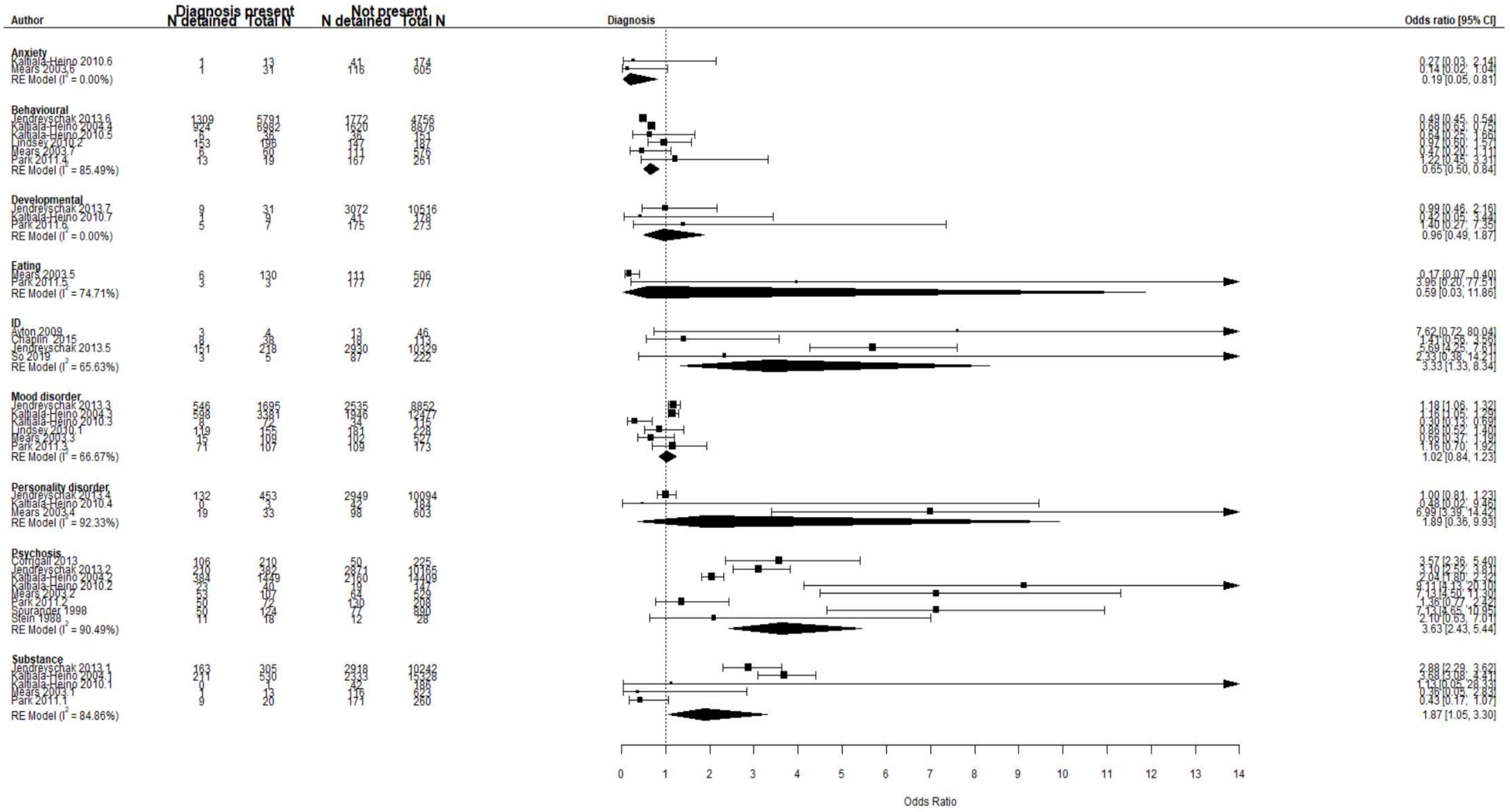

Appendix

Age

Author	Older N detained	Older Total N	Younger N detained	Younger Total N	Odds ratio [95% CI]
**Late adolescence**					
Ellila 2008	39	108	43	170	1.67 [0.99, 2.82]
Sourander 1998	90	396	37	618	4.62 [3.07, 6.94]
Jendreyschak 2013	2884	7893	197	2646	7.16 [6.15, 8.33]
RE Model (I^2 = 88.95%)					2.82 [1.04, 7.63]
**Over 12**					
Lindsey 2010	205	248	59	88	2.34 [1.35, 4.07]
Persi 2016	156	189	24	36	2.36 [1.07, 5.20]
RE Model (I^2 = 90.43%)					3.57 [1.46, 8.73]

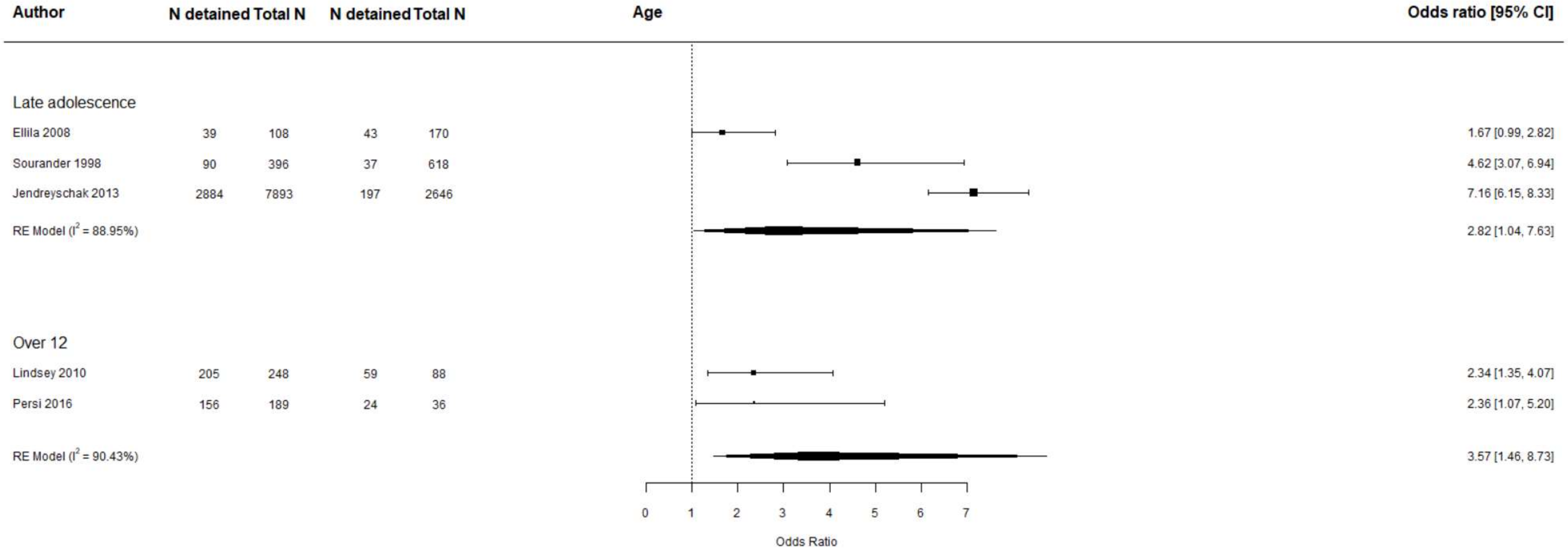

Appendix

Study quality analysis using KMET

Author and year	Question/ Objective sufficiently described	Study design evident and appropriate	Method of subject/ comparison group selection described and appropriate	Subject characteristics sufficiently described	Outcome and exposure measure(s) well defined and robust to measurement/ misclassification bias	Sample size appropriate	Analytic methods described/ justified and appropriate	Some estimate of variance reported for the main results	Controlled for confounding	Results reported in sufficient detail	Conclusions supported by the results	Total	Linear Score	Quality rating
Ayton 2009	1	1	2	2	1	1	2	2	0	1	2	15	68.18	MODERATE
Chaplin 2015	2	2	1	1	2	1	2	2	0	1	2	16	72.72	MODERATE
Corrigall 2013	2	2	1	1	1	1	2	2	0	1	2	15	68.18	MODERATE
Ellila 2008	2	1	1	2	1	2	1	2	2	1	2	17	77.27	HIGH
Jaworowski 1995	1	1	1	1	1	1	1	0	0	1	1	9	40.90	LOW
Jendreyschak 2013	2	2	2	1	2	2	2	2	1	2	2	20	90.90	HIGH
Kaltiala-Heino 2004	2	2	2	1	2	2	2	2	0	2	2	19	86.36	HIGH
Kaltiala-Heino 2010	2	2	2	2	2	1	1	2	2	2	2	20	90.90	HIGH
Khenissi 2004	2	1	2	2	1	1	1	1	0	1	2	14	63.63	MODERATE
Kilgus 1995	1	1	1	1	1	1	1	1	1	2	2	13	59.09	MODERATE
Laget 2002	1	1	1	1	1	1	0	0	0	2	1	9	40.90	LOW
Lindsey 2010	2	2	1	2	2	2	2	2	2	2	2	21	95.45	HIGH
Mears 2003	1	1	1	1	1	1	1	0	0	1	1	9	40.90	LOW
Mertons 2017	1	1	1	1	1		0	1	0	2	1	9	40.90	LOW
Ottisova 2018	2	2	2	2	1	1	2	2	2	2	1	19	86.36	HIGH
Park 2011	1	2	1	2	2	1	1	0	0	1	1	12	54.54	MODERATE
Persi 2016	2	2	2	1	1	2	1	0	0	1	2	14	63.63	MODERATE
Ramel 2015	1	2	2	1	1	1	2	0	0	1	1	12	54.54	MODERATE
Siponen 2007	2	1	2	1	1	2	2	1	0	1	2	15	68.18	MODERATE
So 2019	2	2	1	2	2	1	2	2	2	1	2	19	86.36	HIGH

Appendix

Souranda 1998	1	2	1	1	1	2	2	2	0	1	1	14	63.63	MODERA TE
Stein 1988	1	0	1	0	2	1	1	0	0	1	1	8	36.36	LOW
Tolmac 2004	2	1	2	1	1	1	1	1	0	1	1	12	54.54	MODERA TE

Appendix

Meta-regression: Old (pre-2010) and New (2010-present)

	OR	95% CI	P value
**Intellectual disability**			
Intellectual disability (vs no intellectual disability)	0.8	0.08-7.7	0.85
**Primary Diagnosis**			
Psychosis (vs no psychosis)	0.68	0.24-1.91	0.47
Substance misuse (vs no substance misuse)	0.75	0.01-9.86	0.83
Behavioural Disorder (vs no behavioural disorder)	0.72	0.49-1.06	0.094
Mood Disorder (vs no mood disorder)	0.94	0.47-1.87	0.85
Personality disorder (vs no personality disorder)	0.14	0.07-0.3	<0.0001*
Developmental Disorder	0.89	0.2-4.06	0.88
**Risk**			
Harm to self (vs no harm to self)	0.48	0.13-1.74	0.26
Harm to others (vs no harm to others)	0.74	0.23-2.37	0.62
**Previous admission**			
Previous admission (vs no previous admission)	0.26	0.01-12.69	0.50
**Gender (ref Male)**			
Female	0.68	0.3-1.55	0.36
**Ethnicity (ref White)**			
Black	0.77	0.19-3.15	0.72
**Age**			
Over 12 (vs under 12)	1.79	0.24-13.38	0.57
**Living Arrangements**			
Living with family (vs not living with family)	1.39	0.04-47.76	0.86
**Previous Abuse (ref None)**			
Sexual	0.85	0.04-18.17	0.92

Appendix

Funnel Plots

Gender

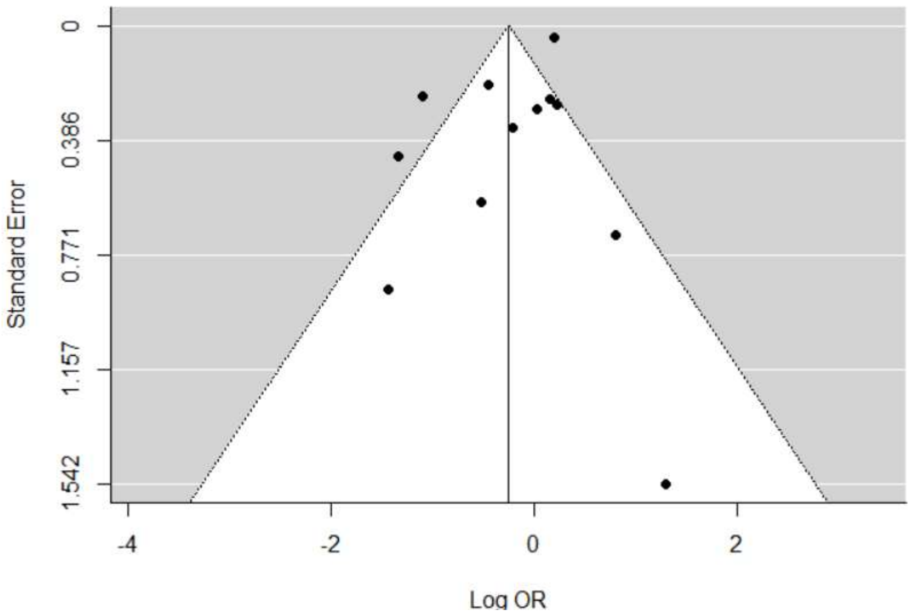

Appendix

Diagnosis

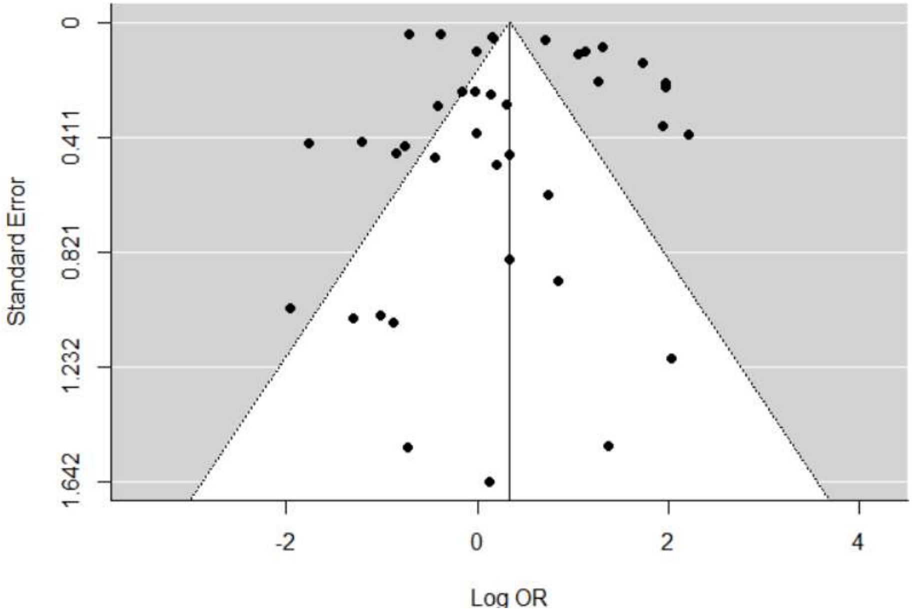

Appendix

Ethnicity

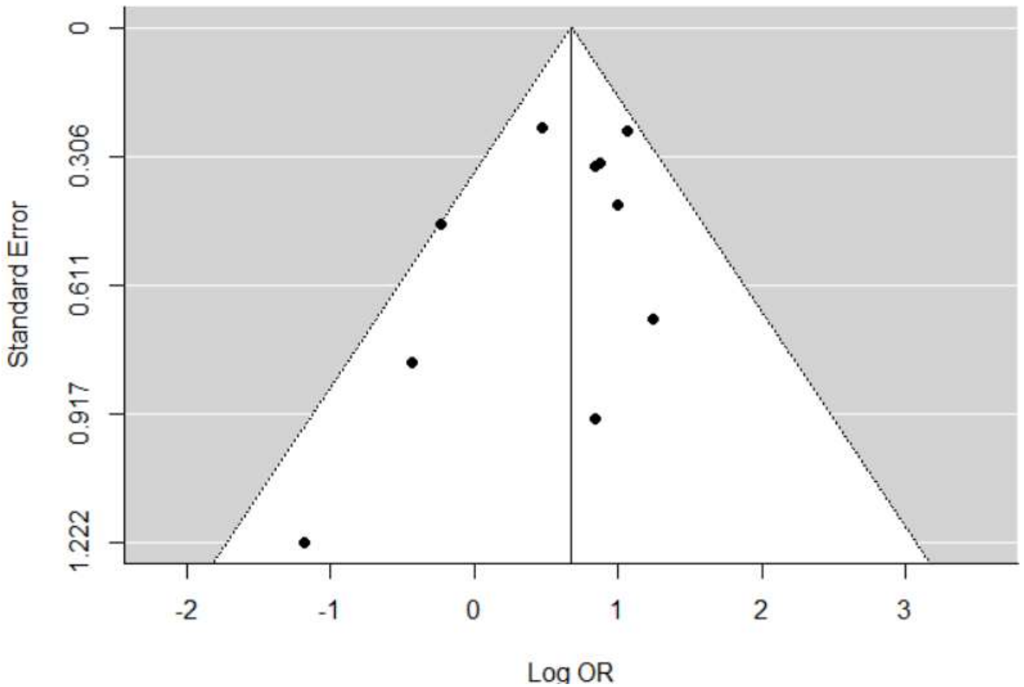

Appendix

Living situation

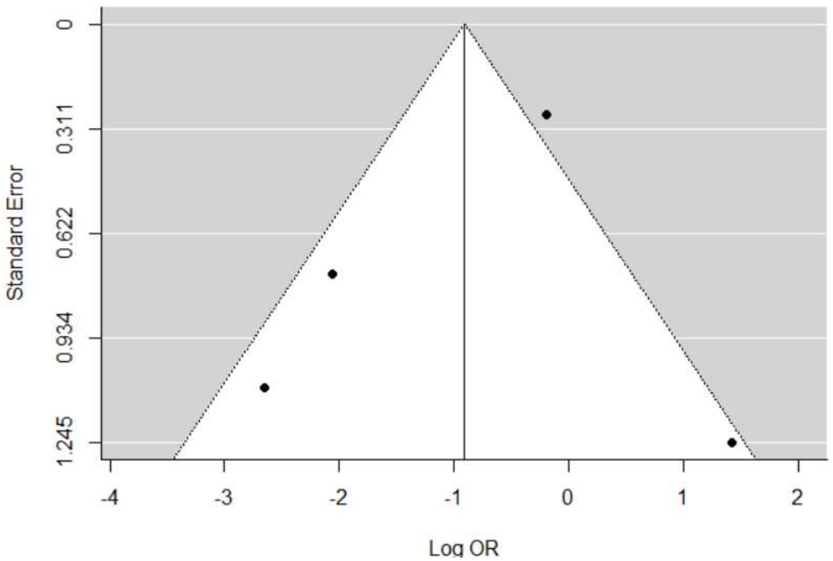

Appendix

Self-harm

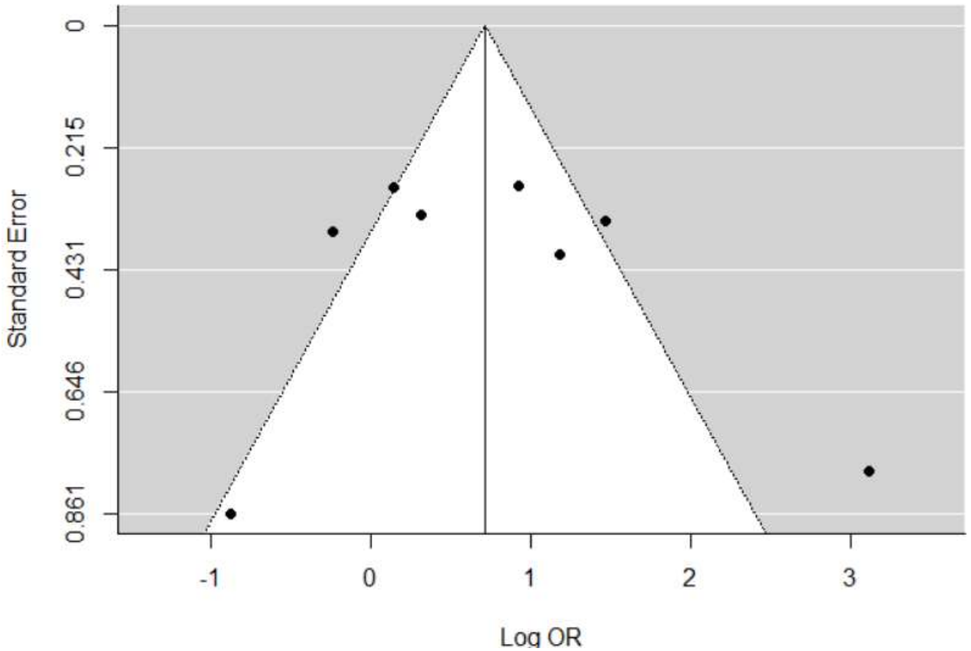

Appendix

Harm to others

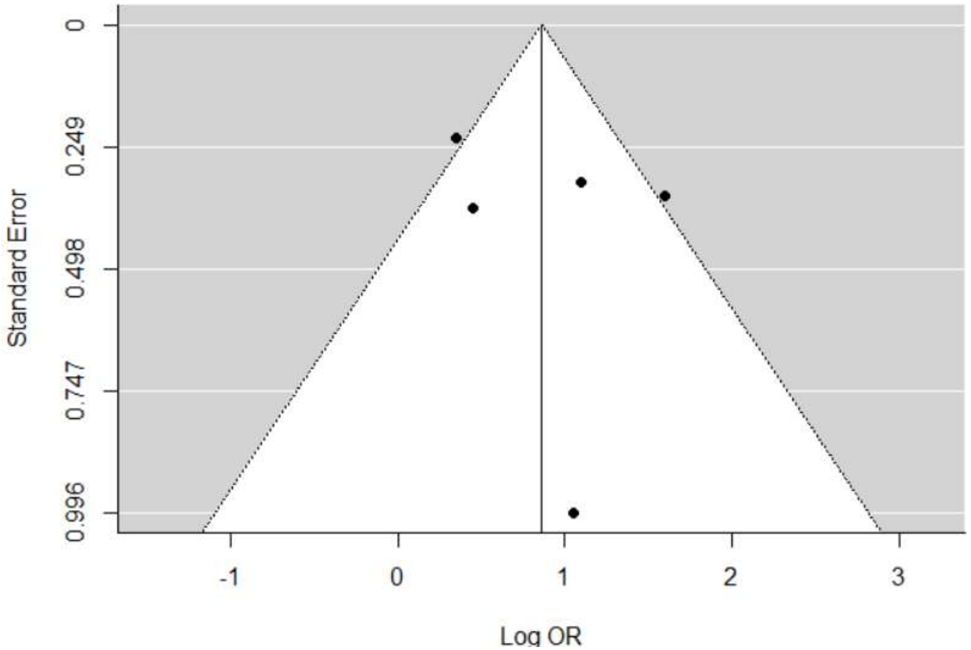

Appendix

Previous abuse

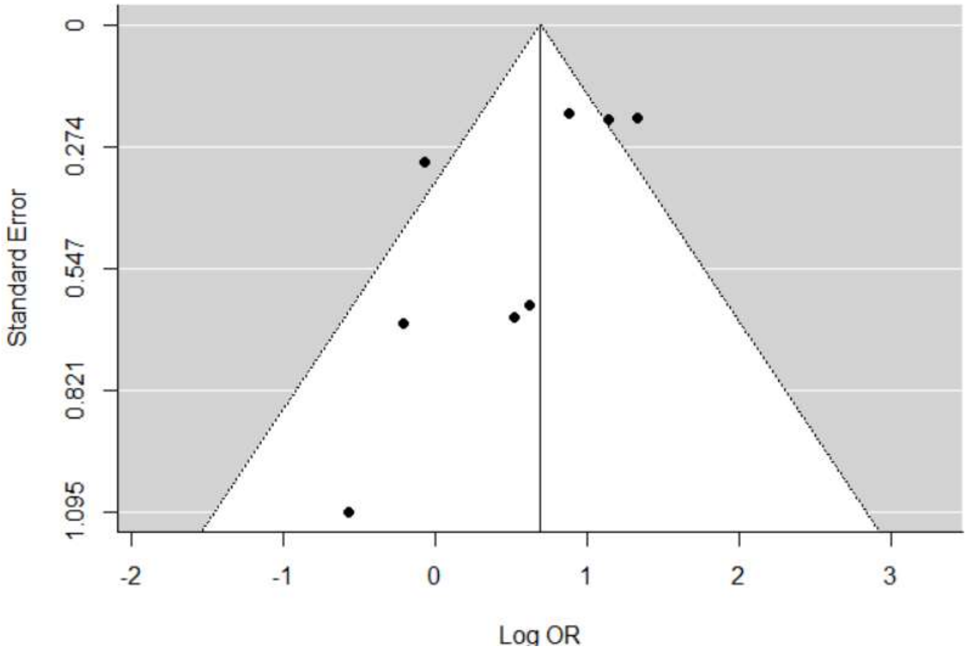

Appendix

Previous admission

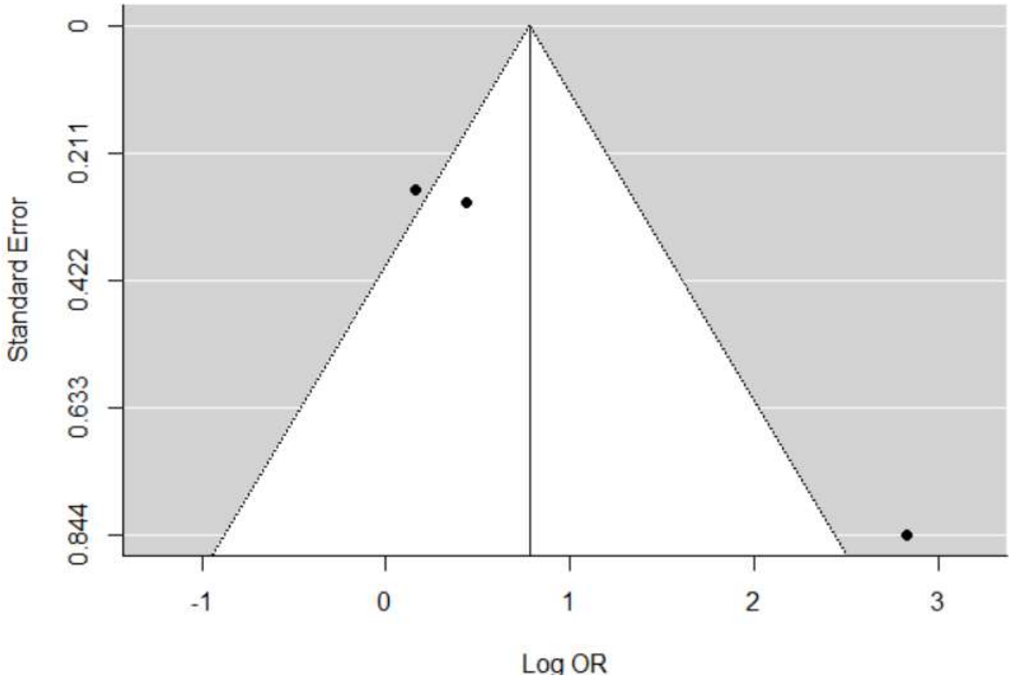

Appendix

Age

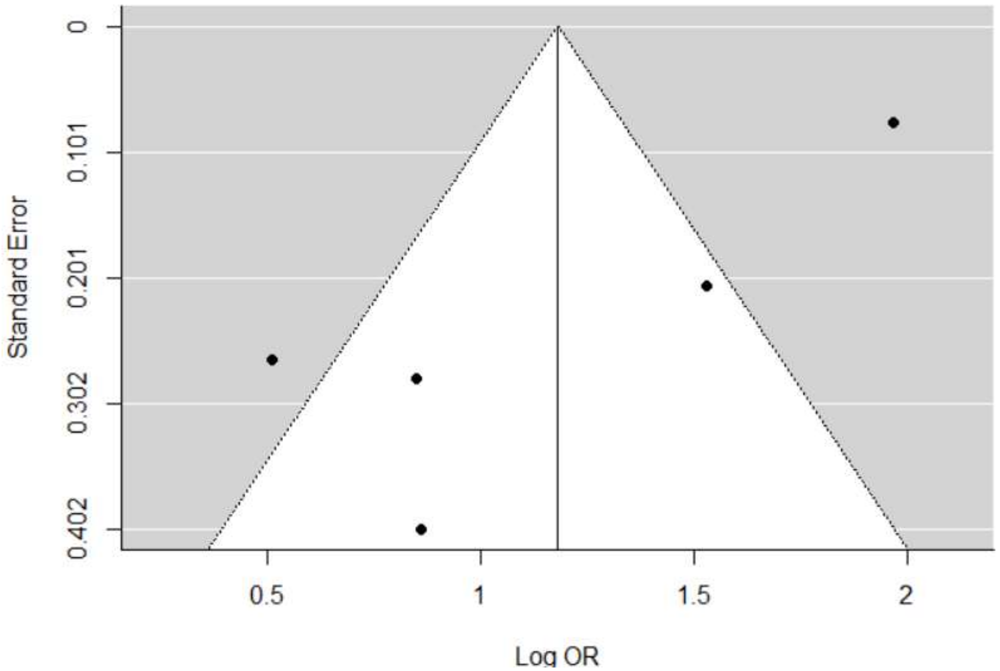

Supplement: Supplementary appendix [file mmc1.pdf]
